# Dual‐Energy CT‐Based Assessment of Thrombotic Heterogeneity for Predicting Stroke Source and Response to Machine Thrombectomy: A Step Toward Visualization Thrombus Treatment

**DOI:** 10.1002/advs.202417295

**Published:** 2025-05-28

**Authors:** Jingxuan Jiang, Sijia Wang, Fan Xiao, Hongmei Gu, Mingkang Wang, Hao Tian, Baohui Guan, Kai Sheng, Yijia Xiong, Huilin Zhao, Minda Li, Li Xu, Zheng Sun, Haiyan Du, Wenxian Du, Yuehua Li

**Affiliations:** ^1^ Institute of Diagnostic and Interventional Radiology Shanghai Sixth People's Hospital Affiliated to Shanghai Jiao Tong University School of Medicine Shanghai 200233 China; ^2^ Department of Radiology Affiliated Hospital of Nantong University Nanton 226001 China; ^3^ Wuhan United Imaging Life Science Instruments Ltd. Wuhan 430206 China; ^4^ Department of Radiology Renji Hospital School of Medicine Shanghai Jiaotong University Shanghai 200001 China; ^5^ Jiangsu Key Laboratory of Integrated Traditional Chinese and Western Medicine for Prevention and Treatment of Senile Diseases Institute of Translational Medicine Medical College Yangzhou University Yangzhou 225001 China

**Keywords:** dual‐energy CT, habitat, radiomics, stroke, thrombus

## Abstract

The viability of using thrombus heterogeneity (TH) data derived from dual‐energy CT (DECT) as a visual thrombotic biomarker is unclear. The first aim of this study is to develop a quantitative measure of TH on DECT and test its performance for predicting the stroke source (cardiogenic vs. non‐cardiogenic) and clinical outcomes (functional status assessed by the modified Rankin Scale score at 90 days) following machine thrombectomy (MT). The second aim is to associate thrombus subregions with the thrombus composition to facilitate visualization of thrombus constituents. Radiomics data are extracted from the whole thrombus and subregions in CT/DECT to construct predictive models. The performances of all models are evaluated and compared in the validation and comparative cohorts. Histopathologic analysis is performed to correlate the subregion data with the actual thrombus composition. This study included 221 and 255 participants who underwent DECT and CT examinations, respectively. DECT outperformed CT in predicting stroke source and clinical outcomes, with the TH‐related models showing the highest performance in the validation and comparative cohorts. Thrombus composition is correlated with the different CT/DECT‐based subregions, with DECT‐habitat_c showing the strongest association. Thrombus subregion analyses may help visualize the related constituents.

## Graphical abstract

The research indicates that dual energy CT scanning can accurately predict the stroke source and clinical outcomes by analyzing thrombus heterogeneity. Based on histopathological analysis of thrombus components, these scans are better able to display thrombus details than standard CT scans, which is helpful for the development of visual navigation thrombectomy to improve stroke management and patient rehabilitation.

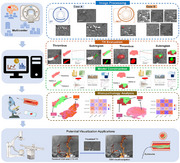

## 1 Introduction

Stroke is a leading cause of adult disability and mortality worldwide, with ischemic stroke accounting for most of all incident strokes in 2019.^[^ ^1^ ^]^ Machine thrombectomy (MT) can help restore blood flow and salvage brain tissue. However, MT outcomes are influenced by numerous clinical factors,^[^ ^2^, ^3^, ^4^, ^5^, ^6^, ^7^, ^8^, ^9^, ^10^ ^]^ and complete recanalization may not be achieved in ≈20% of cases.^[^ ^10^ ^]^ Thrombi with diverse compositions exhibits different susceptibilities to thrombolytic agents and mechanical disruption, leading to varied outcomes post‐MT. Thus, in comparison with red blood cell (RBC)‐rich thrombi, fibrin and platelet (FP)‐rich thrombi are considered resistant to thrombolysis and difficult to retrieve and exhibit a lower degree of responsiveness to revascularization efforts during MT.^[^ ^11^ ^]^ For FP‐rich thrombi, advanced devices are needed to facilitate retrieval with stent retriever,^[^ ^12^ ^]^ and the guidewire should maximally pass through the RBC‐rich area during MT to reduce thrombus escape as a result of fragmentation.

The thrombus heterogeneity (TH) caused by a stroke source within the occluded vessels is being increasingly recognized as a key determinant of thrombus stability.^[^ ^13^ ^]^ Thus, noninvasive and accurate quantification of TH may yield valuable prognostic information and guide personalized treatment strategies. Some special thrombus signs observed on admission imaging have been shown to reflect different compositions and are considered pivotal characteristics in the management and the outcome prediction after MT.^[^ ^7^, ^14^, ^15^ ^]^ Deep learning has revolutionized medical image segmentation, achieving remarkable progress in thrombus segmentation and quantification on computed tomography (CT).^[^ ^16^ ^]^ However, clinical application remains limited due to data dependency, generalizability issues, and computational demands.

The use of radiomics features of thrombi from conventional CT scans is essential for the diagnosis and triage of patients with acute ischemic stroke.^[^ ^17^, ^18^, ^19^ ^]^ However, this approach offers limited insights into the intrinsic properties of thrombi. Dual‐energy CT (DECT), which can distinguish materials on the basis of their energy‐dependent attenuation characteristics, offers a novel window into TH. Multiparametric DECT methods have been used for detection, characterization, and identification of thrombi and for prediction of therapeutic responses in patients with acute ischemic stroke.^[^ ^20^, ^21^, ^22^, ^23^ ^]^ Clustering or voxel‐wise radiomics approaches based on native images or kinetic maps can help depict intratissue heterogeneity,^[^ ^24^, ^25^ ^]^ and integrating these methods with DECT can potentially reveal the spatial distribution and relative proportions of thrombotic constituents.

This study aimed to explore the application of DECT in noninvasive quantification of TH in predicting the stroke source and clinical outcomes following MT, and to correlate the findings with real‐world findings for the thrombotic components. We hypothesize that DECT‐derived metrics of TH can serve as visual thrombotic biomarkers and that the subregions are related to the thrombus components, thereby providing incremental value in thrombus‐related research. Through a comprehensive analysis of CT and DECT images from a large cohort of patients with acute ischemic stroke who underwent MT, we sought to establish a robust prediction model and thrombotic component visualization technique that could stratify patients on the basis of their expected clinical trajectories.

The findings of this study could pave the way for a paradigm shift in stroke management, wherein DECT could become an integral part of the pretreatment imaging workup and thereby enable more informed decision‐making and tailored therapeutic approaches. By harnessing the power of DECT to quantify and even visualize TH, we hope to facilitate the identification of the optimal guidewire pathway for MT.

## 2 Results

### 2.1 Participant Characteristics

**Figure 1:**
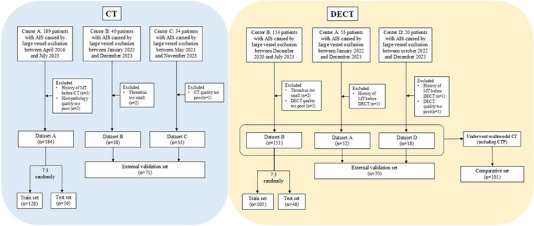
Flowchart showing the process of patient selection for each dataset. CT = computed tomography, DECT = dual‐energy computed tomography, CTP = computed tomography perfusion, AIS = acute ischemic stroke, MT = machine thrombectomy.

A total of 255 participants were included in the CT study: 184 cases from center A randomly divided into training (128 cases) and test (56 cases) sets in a 7:3 ratio, and 38 and 33 cases from centers B and C, respectively, constituting the external validation set. A total of 221 cases were included in the DECT study: 151 cases from center B randomly divided into training (105 cases) and test (46 cases) sets in a 7:3 ratio, and 52 and 18 cases from centers A and D, respectively, constituting the external validation set. Among the patients eligible for the DECT study, 101 cases underwent CT perfusion (CTP) **simultaneously** and were enrolled in the comparative cohort. The patient selection process is presented in **Figure** **1**. The demographic characteristics of the training, testing, and validation cohorts are summarized in **Table** **1**. The demographic characteristics of the subgroups grouped by stroke source and clinical outcomes are summarized in Tables S1 and S2 (Supporting Information).

**Table 1:** Baseline clinical characteristics of the patients.

Characteristics	CT			DECT		
	Train (n = 128)	Test (n = 56)	Validation (n = 71)	Train (n = 105)	Test (n = 46)	Validation (n = 70)
**Age**	72.82±11.84	67.95±11.25	65.95±10.95	70.83±10.67	65.91±11.98	68.39±14.24
**NIHSS**	15.38±8.57	11.41±6.01	10.86±4.98	12.69±6.21	10.83±4.80	10.90±7.16
**Sex**						
Absent	62(48.44%)	19(33.93%)	5(22.73%)	47(44.76%)	16(34.78%)	27(38.57%)
present	66(51.56%)	37(66.07%)	17(77.27%)	58(55.24%)	30(65.22%)	43(61.43%)
**Atrial fibrillation**						
Absent	66(51.56%)	39(69.64%)	17(77.27%)	42(40.00%)	37(80.43%)	47(67.14%)
present	62(48.44%)	17(30.36%)	5(22.73%)	63(60.00%)	9(19.57%)	23(32.86%)
**Smoke**						
Absent	106(82.81%)	39(69.64%)	15(68.18%)	87(82.86%)	34(73.91%)	48(68.57%)
present	22(17.19%)	17(30.36%)	7(31.82%)	18(17.14%)	12(26.09%)	22(31.43%)
**Hypertension**						
Absent	34(26.56%)	11(19.64%)	3(13.64%)	40(38.10%)	17(36.96%)	28(40.00%)
present	94(73.44%)	45(80.36%)	19(86.36%)	65(61.90%)	29(63.04%)	42(60.00%)
**Hyperlipidemia**						
Absent	109(85.16%)	46(82.14%)	16(72.73%)	97(92.38%)	42(91.30%)	51(72.86%)
present	19(14.84%)	10(17.86%)	6(27.27%)	8(7.62%)	4(8.70%)	19(27.14%)
**Diabetes**						
Absent	88(68.75%)	41(73.21%)	17(77.27%)	82(78.10%)	33(71.74%)	51(72.86%)
present	40(31.25%)	15(26.79%)	5(22.73%)	23(21.90%)	13(28.26%)	19(27.14%)
**Coronary disease**						
Absent	102(79.69%)	47(83.93%)	20(90.91%)	97(92.38%)	44(95.65%)	54(77.14%)
present	26(20.31%)	9(16.07%)	2(9.09%)	8(7.62%)	2(4.35%)	16(22.86%)
**Location**						
MCA	89(69.53%)	43(76.79%)	14(63.64%)	75(71.43%)	23(50.00%)	54(77.14%)
ICA	27(21.09%)	9(16.07%)	6(27.27%)	25(23.81%)	18(39.13%)	14(20.00%)
MCA+ICA	12(9.38%)	4(7.14%)	2(9.09%)	5(4.76%)	5(10.87%)	2(2.86%)

Abbreviations: CT = computed tomography, DECT = dual‐energy CT, NIHSS = National Institutes of Health Stroke Scale, MCA = middle cerebral artery, ICA = Internal carotid artery.

### 2.2 Habitat Imaging and Feature Selection

**Figure 2:**
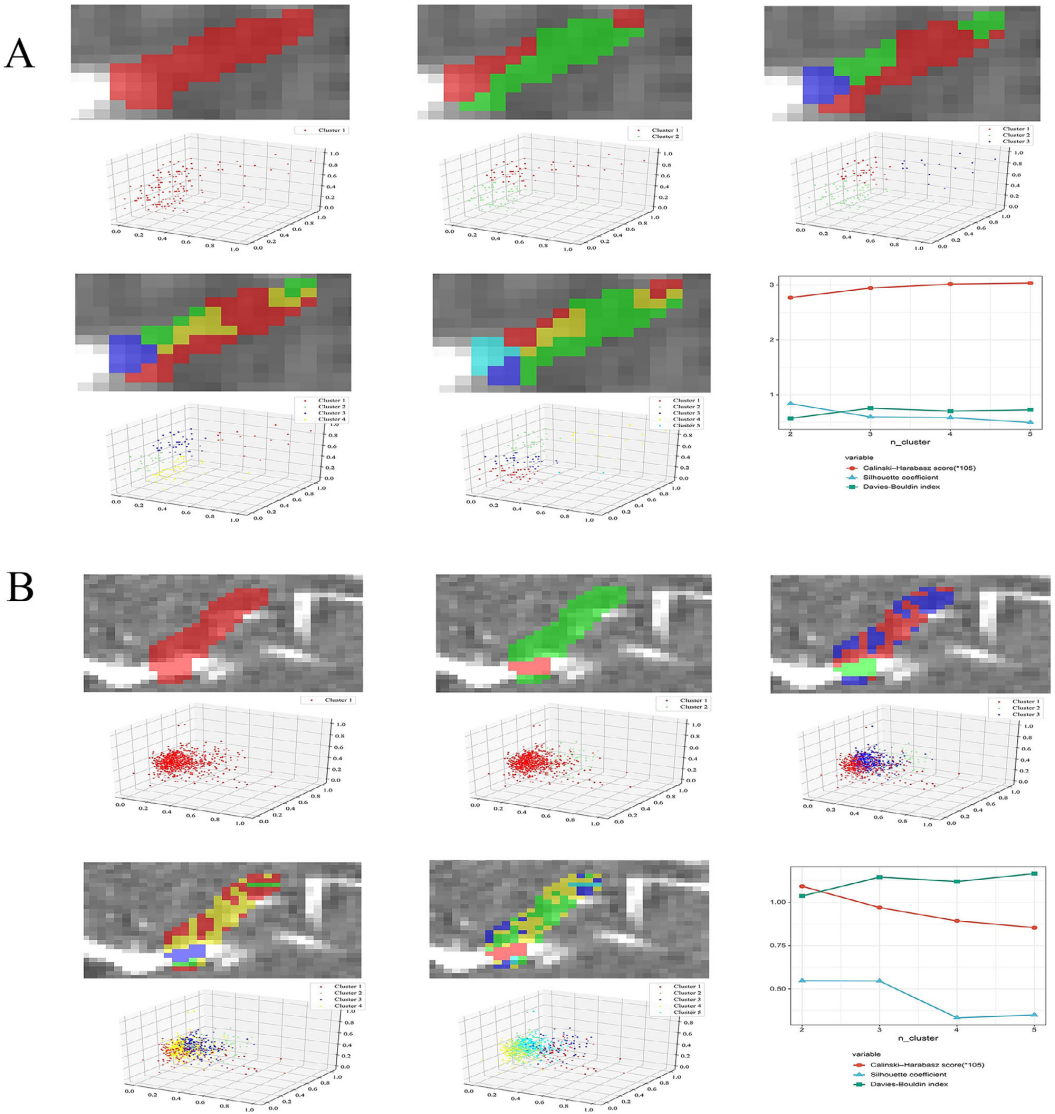
Thrombus clustering was analyzed across 2–5 subregions depicted in CT (A) and DECT (B), the optimal number of clusters was determined to be two. CT = computed tomography, DECT = dual‐energy computed tomography.

**Figure 3:**
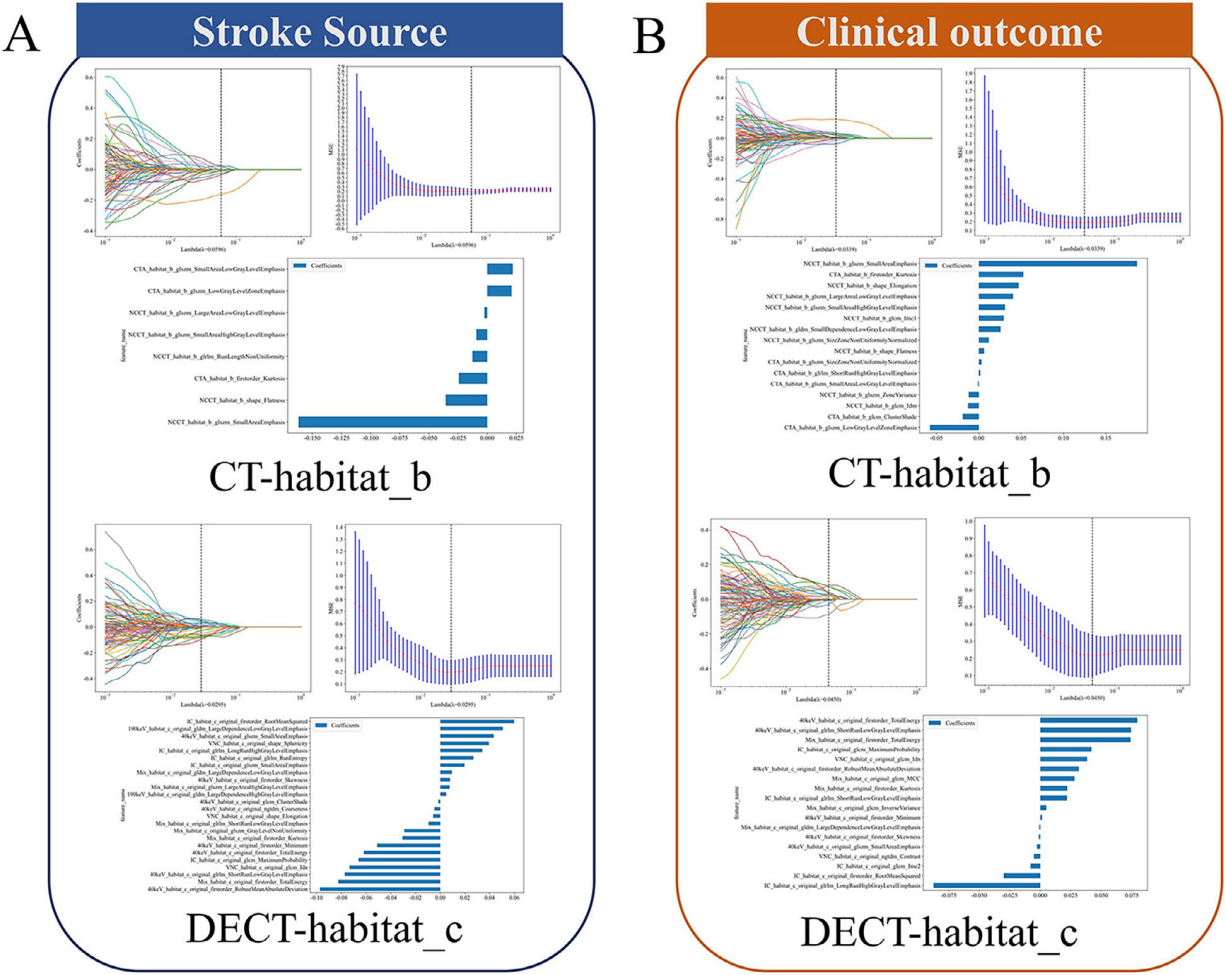
The LASSO regression for feature selection of the CT‐habitat_b and DECT‐habitat_c models to predict stroke source (A) and clinical outcome (B). CT = computed tomography, DECT = dual‐energy computed tomography, LASSO = least absolute shrinkage and selection operator.

To determine the optimal number of clusters for thrombus subregion analysis, we evaluated clustering configurations using the Calinski‐Harabasz score, Silhouette coefficient, and Davies‐Bouldin index. These metrics were applied to assess the balance between within‐cluster similarity and between‐cluster separation. The Calinski‐Harabasz score measured the ratio of between‐cluster variance to within‐cluster variance, with higher scores indicating better‐defined clusters. The Silhouette coefficient evaluated the average similarity of each data point to its assigned cluster, with values closer to 1 indicating stronger clustering. The Davies‐Bouldin index assessed the average similarity between each cluster and its most similar cluster, with lower values indicating better clustering quality. After testing configurations ranging from 2 to 5 clusters, the optimal number of clusters was determined to be two. This configuration, consisting of **habitat_a and habitat_b** in the CT study (**Figure** **2**A), as well as **habitat_c and habitat_d** in the DECT study (Figure 2B), effectively balanced within‐cluster similarity and between‐cluster separation, ensuring robust clustering performance. A total of 107 features were extracted from each CT sequence, after feature reduction and least absolute shrinkage and selection operator (LASSO) selection, 4–36 features were retained in the radiomics or habitat models based on CT/DECT to predict stroke source and clinical outcomes. The LASSO regression for feature selection of CT‐habitat_b and DECT‐habitat_c to predict stroke source and clinical outcome is shown in **Figure** **3**A,B, while the remaining selections are presented in Figure S1 (Supporting Information).

### 2.3 Development and Testing of the Predictive Models

#### 2.3.1 Performance of the Different Machine Learning Algorithms

**Figure 4:**
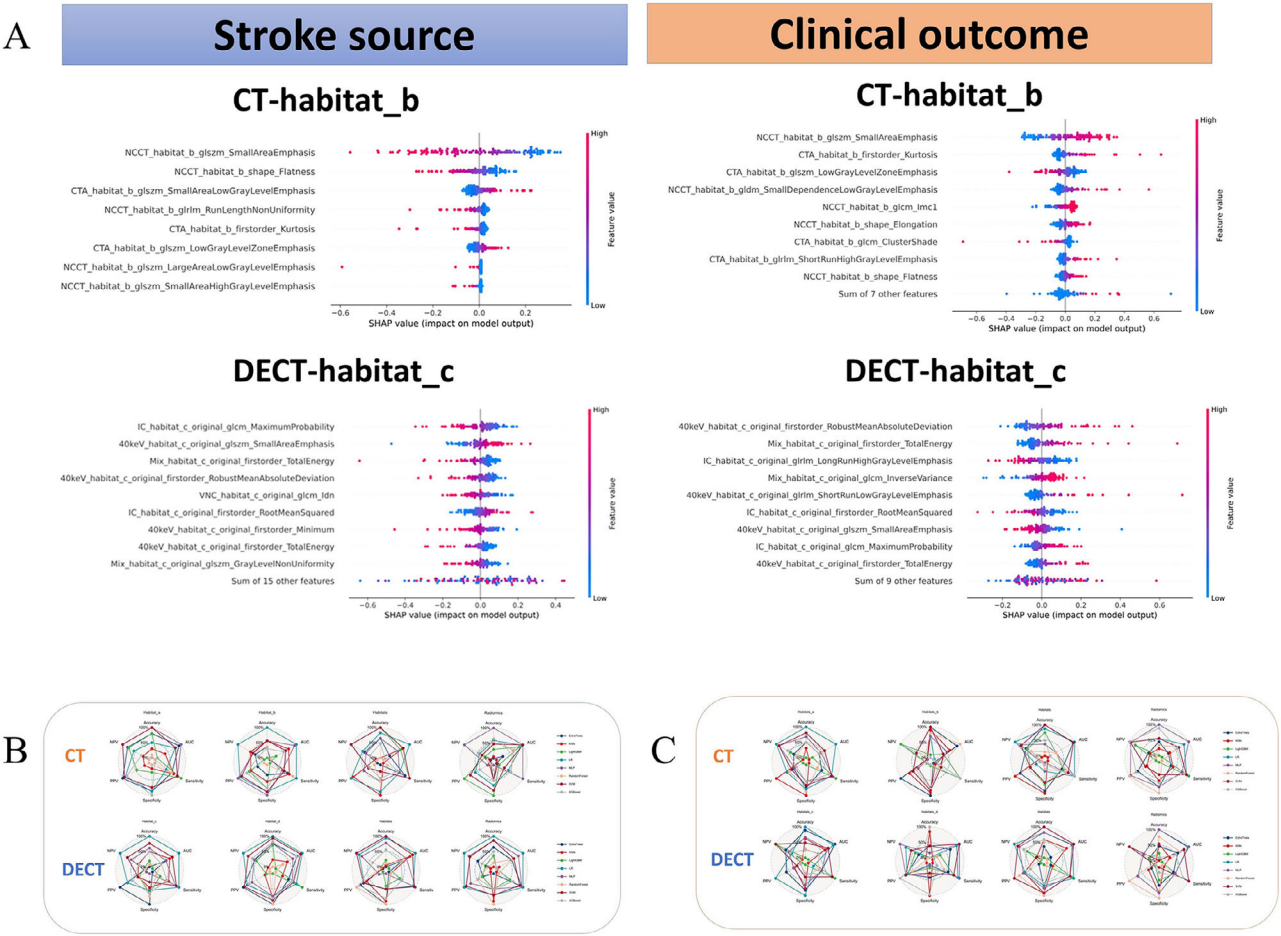
SHAP summary dot plot of logistic regression algorithms in predicting stroke source and clinical outcomes based on CT‐habitat_b and DECT‐habitat_c (A). The radar charts show AUC, accuracy, sensitivity, specificity, NPV, and PPV of different models in predicting the stroke source (B) and clinical outcome (C). LR model demonstrated the largest comprehensive area, indicating its superior performance. SHAP = shapely additive explanation, CT = computed tomography, DECT = dual‐energy CT, AUC = area under the curve of the receiver operating characteristic curve, PPV = positive predictive values, NPV = negative predictive values, LR = logistic regression.

The results of fivefold cross‐validation indicated that the optimal machine learning algorithms to build signatures for the radiomics and TH models were random forest, logistic regression, support vector machine, k‐nearest neighbor, extra trees, light gradient boosting machine, multi‐layer perceptron, and extreme gradient boosting. The shapely additive explanation (SHAP) summary dot plot for logistic regression algorithms predicting stroke source and clinical outcomes based on CT‐habitat_b and DECT‐habitat_c is shown in **Figure** **4**A, while the remaining plots are presented in Figure S2 (Supporting Information). Logistic regression was ultimately chosen for the final model. In the validation set, it achieved a maximum area under the receiver operating characteristic curve (AUC) of 0.869 and a maximum Matthews correlation coefficient (MCC) of 0.823. Tables S3 and S4 (Supporting Information) and Figure 4B,C show the performance of the different models.

#### 2.3.2 Performance of the Different Models Based on LR Algorithms

**Figure 5:**
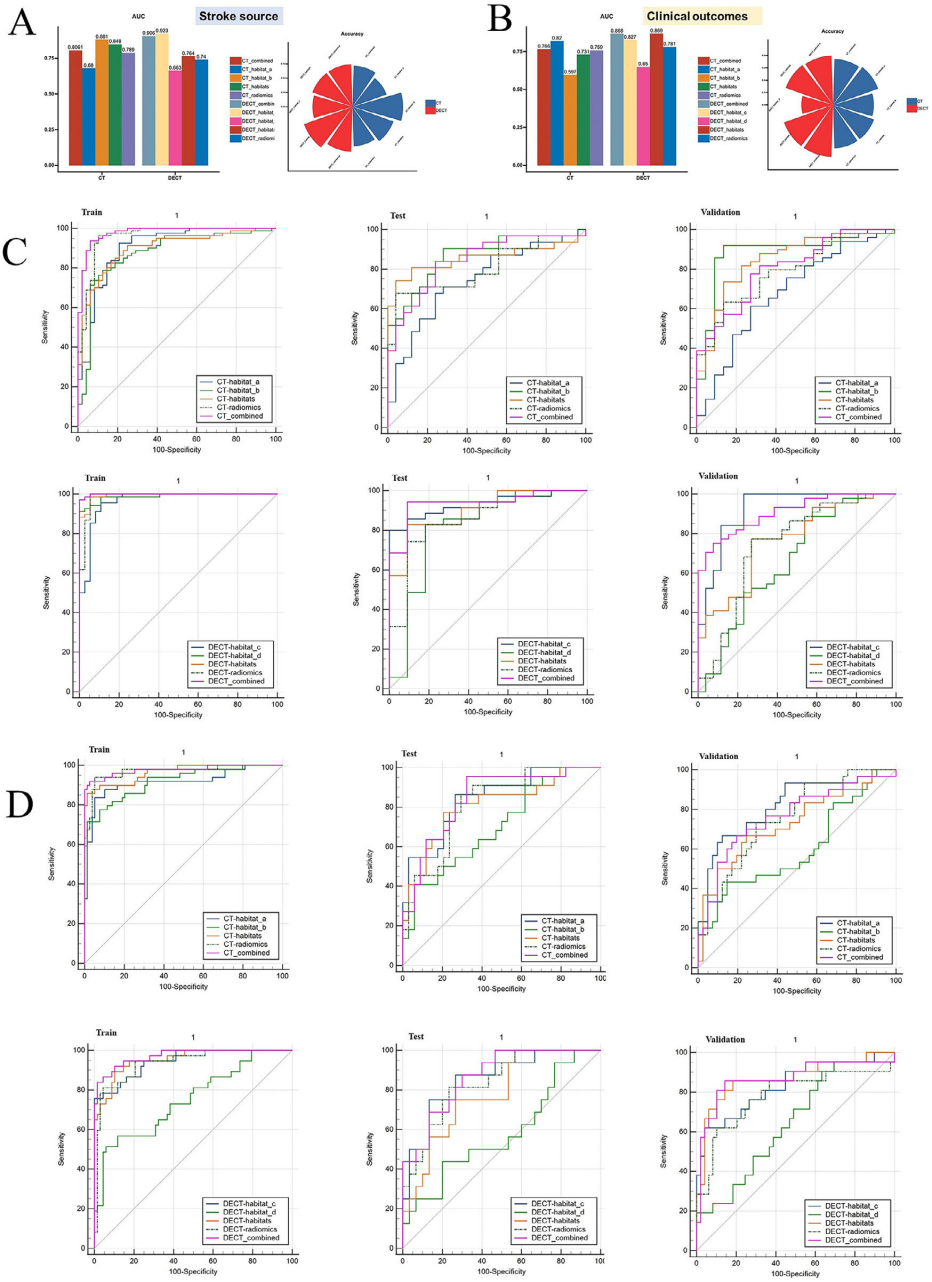
The performance of different models in predicting stroke source (A) and clinical outcomes (B) based on CT/DECT scans in the validation set (CT: n = 71; DECT: n = 70). The ROC curves for predicting the stroke source (C) and clinical outcome (D) based on CT and DECT thrombus and habitats in train (CT: n = 128; DECT: n = 105), test (CT: n = 56; DECT: n = 46) and validation (CT: n = 71; DECT: n = 70) sets. The value of AUCs and the accuracy of the models based on DECT (AUC_max_: 0.923 & 0.869, Accuracy_max_: 0.900 & 0.843) were higher than those based on CT (AUC_max_: 0.881 & 0.820, Accuracy_max_: 0.887 & 0.775) in the validation cohort (CT: n = 71; DECT: n = 70). CT = computed tomography, DECT = dual‐energy CT, ROC = receiver operating characteristic curve, AUC = area under the curve of the ROC.

In the validation cohort, when the stroke source was predicted on the basis of CT scans, the AUCs of the radiomics, habitat_a, habitat_b, habitats, and combined models were 0.789 (0.682–0.897), 0.680 (0.542‐0.818), 0.881 (0.787‐0.975), 0.848 (0.750–0.946), and 0.806 (0.714–0.898), respectively. The prediction performances of the habitat_b, habitats, and combined models were higher than that of the habitat_a model (p = 0.009, 0.019, and 0.005, respectively), with no significant difference between the radiomics model and the habitat_a, habitat_b, habitats, and combined models (p = 0.075, 0.185, 0.312, and 0.539, respectively). In the analyses based on DECT scans, the AUCs of the radiomics, habitat_c, habitat_d, habitats and combined models were 0.740 (0.609–0.872), 0.923 (0.853–0.993), 0.663 (0.522–0.803), 0.764 (0.650–0.879), and 0.907 (0.838–0.975), respectively. The prediction performances of the habitat_c and combined models were higher than those of the habitat_d (all *p* < 0.001), habitats (all *p* < 0.01), and radiomics models (*p* = 0.025 and 0.004). When the clinical outcomes were predicted on the basis of CT scans, the AUCs of the radiomics, habitat_a, habitat_b, habitats, and combined models were 0.759 (0.648–0.870), 0.820 (0.719–0.922), 0.597 (0.458–0.736), 0.731 (0.608–0.854), and 0.766 (0.667–0.865), respectively. The prediction performances of the habitat_a, habitats, radiomics, and combined models were lower than that of the habitat_b model (*p* = 0.005, 0.002, 0.032, and 0.004, respectively), but no significant difference was observed between the radiomics model and the habitat_a, habitats, and combined models (*p* = 0.319, 0.659, and 0.847, respectively). When the clinical outcomes were predicted on the basis of DECT scans, the AUCs of the radiomics, habitat_c, habitat_d, habitats, and combined models were 0.781 (0.643–0.920), 0.827 (0.709–0.945), 0.650 (0.513–0.787), 0.869 (0.762–0.975), and 0.868 (0.789–0.947), respectively. The prediction performance of the combined model was higher than that of the radiomics model (*p* = 0.025), and those of the habitats and combined models were higher than that of the habitat_d model (p = 0.005 and 0.009, respectively). More details regarding the performance of different models in predicting stroke source and clinical outcomes are presented in **Tables** **2** and **3**. The AUC and accuracy values of the DECT‐based models (AUC_max_: 0.923 & 0.869, Accuracy_max_: 0.900 & 0.843) were higher than those of the CT‐based models (AUC_max_: 0.881 & 0.820, Accuracy_max_: 0.887 & 0.775) in the validation cohort (**Figure** **5**).

**Table 2:** The performance of different models in predicting stroke source.

Models	Accuracy	AUC	95% CI	Sensitivity	Specificity	PPV	NPV	Threshold
**CT_habitat_a‐train**	0.867	0.904	0.846 – 0.962	0.912	0.792	0.880	0.844	0.585
**CT_habitat_a‐test**	0.696	0.743	0.612 – 0.874	0.645	0.760	0.769	0.633	0.704
**CT_habitat_a‐val**	0.634	0.680	0.542 – 0.818	0.592	0.727	0.829	0.444	0.663
**CT_habitat_b‐train**	0.812	0.874	0.807 – 0.942	0.775	0.875	0.912	0.700	0.663
**CT_habitat_b‐test**	0.804	0.867	0.772 – 0.962	0.871	0.720	0.794	0.818	0.607
**CT_habitat_b‐val**	0.887	0.881	0.787 – 0.975	0.898	0.864	0.936	0.792	0.506
**CT_habitats‐train**	0.828	0.899	0.844 – 0.953	0.837	0.812	0.882	0.750	0.595
**CT_habitats‐test**	0.821	0.854	0.745 – 0.963	0.710	0.960	0.957	0.727	0.728
**CT_habitats‐val**	0.761	0.848	0.750– 0.946	0.714	0.864	0.921	0.576	0.682
**CT_radiomics‐train**	0.930	0.957	0.920– 0.995	0.950	0.896	0.938	0.915	0.573
**CT_radiomics‐test**	0.786	0.804	0.687 – 0.921	0.645	0.960	0.952	0.686	0.819
**CT_radiomics‐val**	0.690	0.789	0.682 – 0.897	0.612	0.864	0.909	0.500	0.866
**CT_combined‐train**	0.922	0.978	0.953 – 1.000	0.938	0.896	0.938	0.938	1.312
**CT_combined‐test**	0.732	0.845	0.750‐0.940	0.677	0.800	0.808	0.667	−0.920
**CT_combined‐val**	0.718	0.806	0.714 – 0.898	0.837	0.455	0.556	0.774	6.415
**DECT_habitat_c‐train**	0.924	0.964	0.927 – 1.000	0.941	0.892	0.941	0.892	0.535
**DECT_habitat_c‐test**	0.826	0.930	0.859 – 1.000	0.771	1.000	1.000	0.579	0.762
**DECT_habitat_c‐val**	0.900	0.923	0.853 – 0.993	0.977	0.769	0.878	0.952	0.509
**DECT_habitat_d‐val**	0.700	0.663	0.522 – 0.803	0.841	0.462	0.725	0.632	0.230
**DECT_habitat_d‐train**	0.933	0.988	0.974 – 1.000	0.897	1.000	1.000	0.841	0.692
**DECT_habitat_d‐test**	0.804	0.816	0.635 – 0.996	0.800	0.818	0.933	0.562	0.425
**DECT_habitats‐train**	0.962	0.993	0.983 – 1.000	0.971	0.946	0.971	0.946	0.606
**DECT_habitats‐test**	0.826	0.901	0.806 – 0.997	0.800	0.909	0.966	0.588	0.437
**DECT_habitats‐ val**	0.743	0.764	0.650 – 0.879	0.750	0.731	0.825	0.633	0.683
**DECT_radiomics‐train**	0.971	0.986	0.964 – 1.000	0.985	0.946	0.971	0.972	0.590
**DECT_radiomics‐test**	0.761	0.852	0.718 – 0.986	0.714	0.909	0.962	0.500	0.649
**DECT_radiomics‐val**	0.743	0.740	0.609‐ 0.872	0.750	0.731	0.825	0.633	0.633
**DECT_combined‐train**	0.971	0.999	0.992 – 1.000	0.985	0.946	0.971	0.972	1.343
**DECT_combined‐test**	0.739	0.938	0.868 – 1.000	0.686	0.909	0.960	0.476	−6.396
**DECT_combined‐val**	0.786	0.907	0.838 ‐0.975	0.977	0.462	0.923	0.754	13.576

Abbreviations: CT = computed tomography, DECT = dual‐energy CT, AUC = area under the curve of the receiver operating characteristic curve, CI = confidence interval, PPV = positive predictive values, NPV = negative predictive values.

**Table 3:** The performance of different models in predicting Clinical outcomes.

Models	Accuracy	AUC	95% CI	Sensitivity	Specificity	PPV	NPV	Threshold
**CT_habitat_a‐train**	0.898	0.919	0.860 – 0.977	0.816	0.949	0.909	0.893	0.497
**CT_habitat_a‐val**	0.775	0.820	0.719 – 0.922	0.633	0.878	0.792	0.766	0.563
**CT_habitat_a‐test**	0.768	0.845	0.741 – 0.949	0.818	0.735	0.667	0.862	0.317
**CT_habitat_b‐train**	0.875	0.914	0.862 – 0.967	0.694	0.987	0.971	0.839	0.648
**CT_habitat_b‐test**	0.714	0.702	0.561 – 0.843	0.364	0.941	0.800	0.696	0.829
**CT_habitat_b‐val**	0.662	0.597	0.458 – 0.736	0.400	0.854	0.667	0.660	0.693
**CT_habitats‐train**	0.930	0.964	0.933 – 0.994	0.837	0.987	0.976	0.907	0.503
**CT_habitats‐test**	0.768	0.809	0.687 – 0.931	0.727	0.794	0.696	0.818	0.445
**CT_habitats‐val**	0.704	0.731	0.608 – 0.854	0.633	0.756	0.655	0.738	0.374
**CT_radiomics‐train**	0.938	0.968	0.937 – 0.999	0.918	0.949	0.918	0.949	0.401
**CT_radiomics‐val**	0.704	0.759	0.648 – 0.870	0.700	0.707	0.636	0.763	0.348
**CT_radiomics‐test**	0.750	0.817	0.706 – 0.928	0.818	0.706	0.643	0.857	0.531
**CT_combined‐train**	0.953	0.977	0.950 ‐1.000	0.878	1.000	1.000	0.929	−0.061
**CT_combined‐test**	0.750	0.844	0.748 – 0.939	0.773	0.735	0.654	0.833	−0.162
**CT_combined‐val**	0.747	0.766	0.667 ‐0.865	0.227	0.265	0.346	0.167	7.656
**DECT_habitat_c‐train**	0.905	0.946	0.905 – 0.987	0.730	1.000	1.000	0.872	0.592
**DECT_habitat_c‐test**	0.804	0.854	0.739 – 0.969	0.687	0.867	0.733	0.839	0.571
**DECT_habitat_c‐val**	0.843	0.827	0.709 – 0.945	0.571	0.959	0.857	0.839	0.670
**DECT_habitat_d‐train**	0.781	0.751	0.650 – 0.852	0.486	0.941	0.818	0.771	0.462
**DECT_habitat_d‐test**	0.543	0.417	0.234 – 0.599	0.312	0.667	0.333	0.645	0.417
**DECT_habitat_d‐val**	0.514	0.650	0.513 – 0.787	0.857	0.367	0.367	0.857	0.256
**DECT_habitats‐train**	0.886	0.953	0.915 – 0.991	0.865	0.897	0.821	0.924	0.381
**DECT_habitats‐val**	0.814	0.869	0.762 – 0.975	0.810	0.816	0.654	0.909	0.412
**DECT_habitats‐test**	0.717	0.779	0.642 – 0.916	0.687	0.733	0.579	0.815	0.368
**DECT_radiomics‐train**	0.895	0.936	0.887 – 0.984	0.784	0.956	0.906	0.890	0.523
**DECT_radiomics‐test**	0.761	0.838	0.720 – 0.955	0.750	0.767	0.632	0.852	0.304
**DECT_radiomics‐val**	0.800	0.781	0.643 – 0.920	0.571	0.898	0.706	0.830	0.688
**DECT_combined‐train**	0.924	0.971	0.940‐1.000	0.811	0.985	0.968	0.905	0.401
**DECT_combined‐test**	0.783	0.867	0.768‐0.965	0.625	0.867	0.714	0.813	−1.827
**DECT_combined‐val**	0.843	0.868	0.788‐0.947	0.714	0.898	0.880	0.750	6.276

Abbreviations: CT = computed tomography, DECT = dual‐energy CT, AUC = area under the curve of the receiver operating characteristic curve, CI = confidence interval, PPV = positive predictive values, NPV = negative predictive values.

### 2.4 Association of the Intra‐Thrombus Subregions with the Thrombus Composition

**Figure 6:**
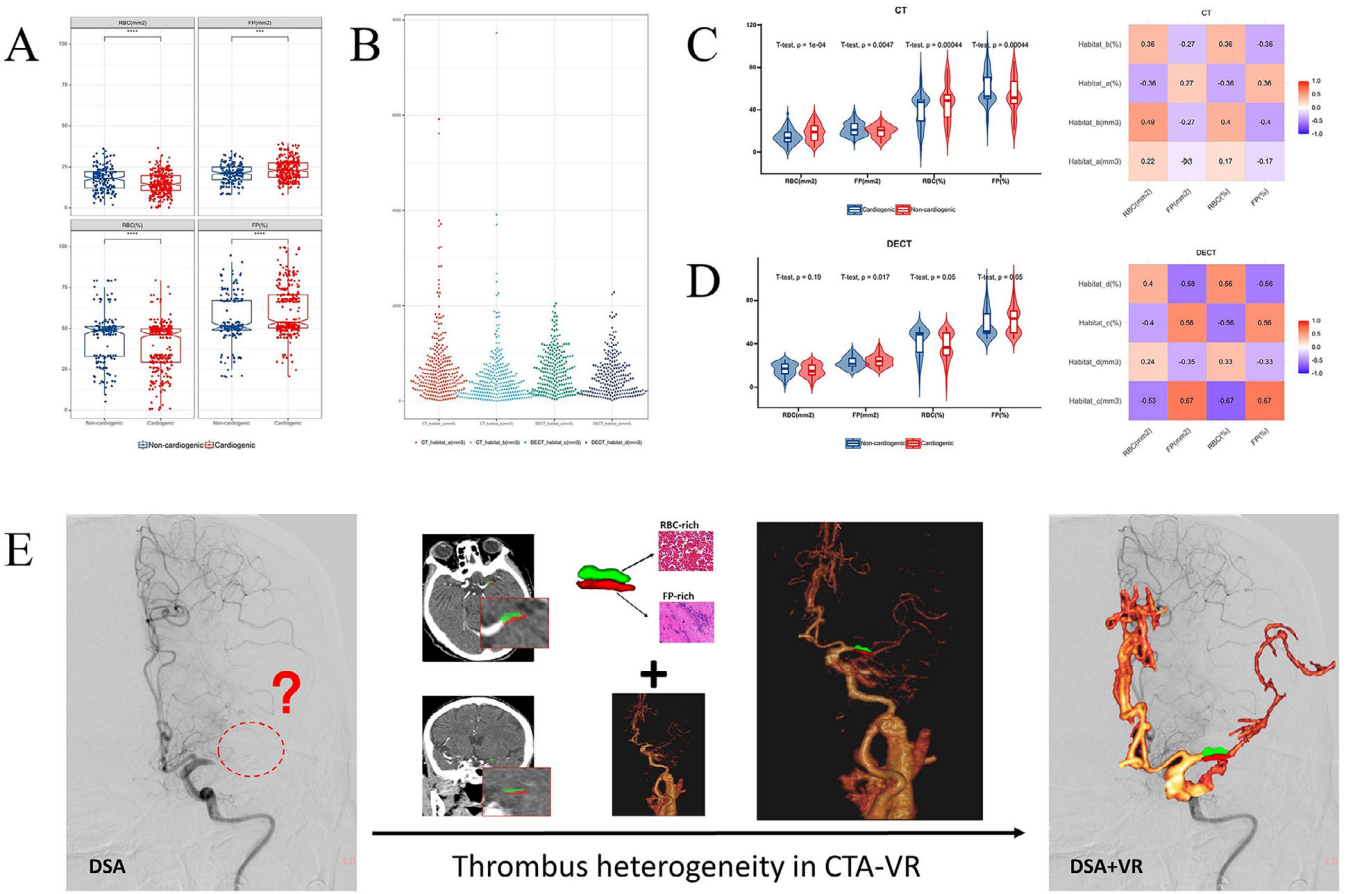
Results of histological analysis. In comparison with non‐cardiogenic thrombi (n = 169), the cardiogenic thrombi (n = 307) showed a significantly higher area and proportion of FP and lower area and proportion of RBCs (A). The distribution of different subregion volumes in CT (n = 255) and DECT (n = 221) (B). Pearson correlation coefficients showed that two main thrombus constituents correlated with the subregions based on CT (C), with lower correlation coefficient values than those for DECT (D). Thrombus component visualization to guide thrombectomy (E). CT = computed tomography, DECT = dual‐energy CT, CTA = computed tomography angiography, RBC = red blood cell, FP = fibrin and platelet, DSA = digital subtraction angiography, VR = **virtual reality**.

We performed histological analysis of thrombi collected from all patients. In comparison with the non‐cardiogenic thrombi, the cardiogenic thrombi showed significantly higher area (23.15 ± 6.08 mm^2^ vs. 21.00 ± 5.71 mm^2^, *p* < 0.001) and proportion of FP (61.20 ± 14.78% vs. 55.18 ± 15.64%, p < 0.001) and significantly lower area (14.91 ± 6.50 mm^2^ vs. 17.57 ± 7.19 mm^2^, *p* < 0.001) and proportion of RBCs (38.80 ± 14.78% vs. 44.84 ± 15.64%, *p* < 0.001). Pearson correlation coefficient analysis showed that the two main thrombus constituents (RBCs and FP) were correlated with the subregions based on DECT (|rmax| = 0.666, *p* < 0.001), and this correlation was higher than that for CT (|rmax| = 0.487, *p* < 0.001). In addition, the volume of DECT‐habitat_c showed the strongest association with the area of the histopathological components (rRBCs = ‐0.526; rFP = 0.666) (**Figure** **6**).

### 2.5 Model Comparison and Histological Analysis in the Comparative Cohort

**Figure 7:**
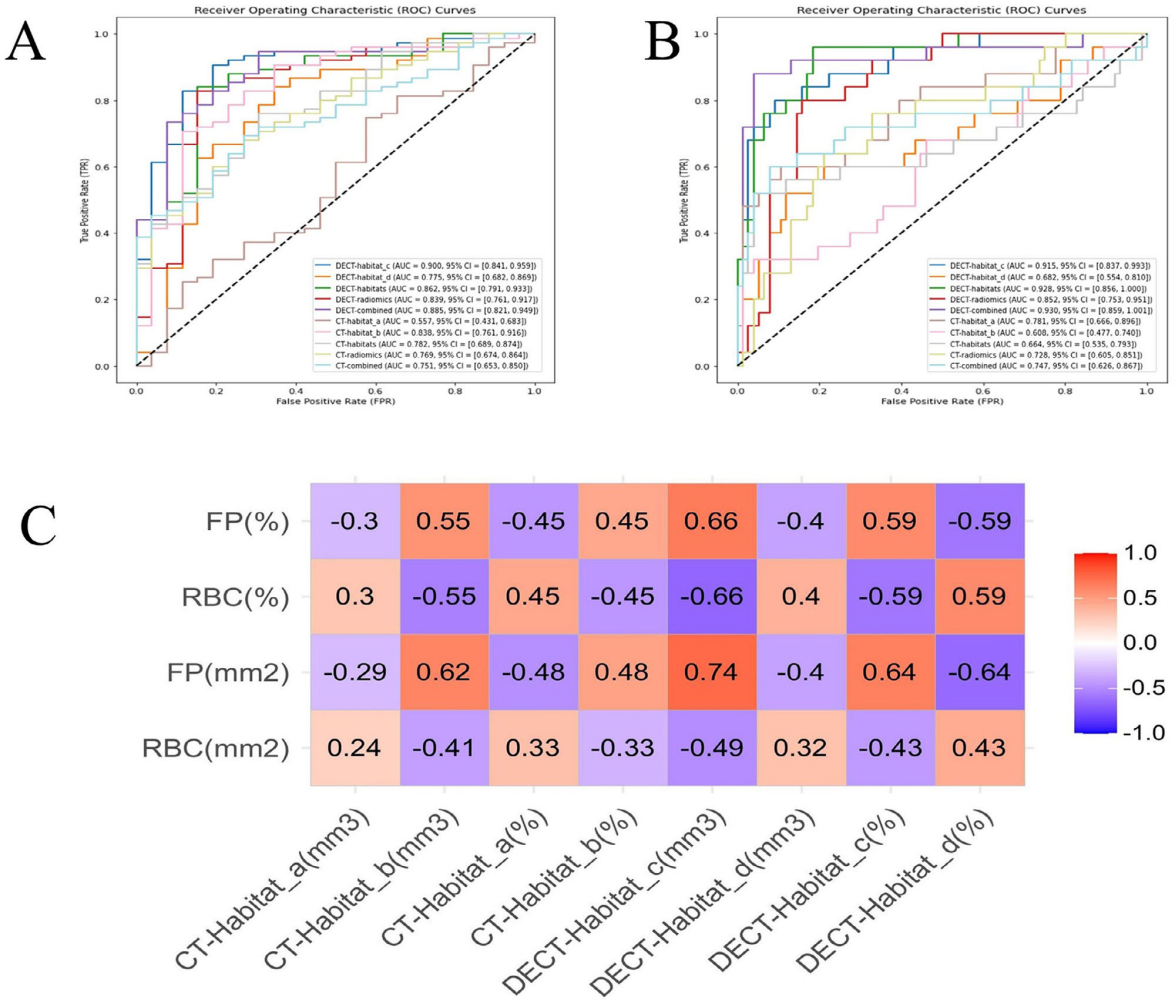
The ROC curves for predicting the stroke source (A) and clinical outcome (B) based on CT and DECT thrombus and habitats in comparative set (n = 101). Pearson correlation coefficients showed that two main thrombus constituents correlated with the subregions based on CT and DECT (C). CT = computed tomography, DECT = dual‐energy CT, RBC = red blood cell, FP = fibrin and platelet.

In the comparative cohort, the AUCs for predicting stroke source were: CT‐habitat_a, 0.557 (0.431–0.683); CT‐habitat_b, 0.838 (0.761–0.916); CT‐habitats, 0.782 (0.689–0.874); CT‐radiomics, 0.769 (0.674–0.864); and CT‐combined, 0.751 (0.653–0.850); DECT‐habitat_c, 0.900 (0.841–0.959); DECT‐habitat_d, 0.775 (0.682–0.869); DECT‐habitats, 0.862 (0.791–0.933); DECT‐radiomics, 0.839 (0.761–0.917); DECT‐combined, 0.885 (0.821–0.949). The DeLong test results showed significant differences in several comparisons. DECT‐habitat_c had significant differences compared to CT‐habitat_a (*p* < 0.001) and CT‐combined (*p* = 0.011), but no significant difference was observed between DECT‐habitat_c and CT‐habitat_b (*p* = 0.217) (**Figure** **7**A). The AUCs for predicting clinical outcomes were: CT‐habitat_a, 0.781 (0.666–0.896); CT‐habitat_b, 0.608 (0.477–0.740); CT‐habitats, 0.664 (0.535–0.793); CT‐radiomics, 0.728 (0.605–0.851); CT‐combined, 0.747 (0.626–0.867); DECT‐habitat_c, 0.915 (0.837–0.993); DECT‐habitat_d, 0.682 (0.554–0.810); DECT‐habitats, 0.928 (0.856–1.000); DECT‐radiomics, 0.852 (0.753–0.951); and DECT‐combined, 0.930 (0.859–1.000). The DeLong test results reveal significant differences between DECT‐habitat_c and DECT‐habitat_d (*p* = 0.002). DECT‐habitat_c also showed significant differences compared to several CT‐based methods (*p* < 0.05) (Figure 7B). Pearson correlation coefficient analysis showed that the two main thrombus constituents (RBCs and FP) were correlated with the subregions based on DECT (|rmax| = 0.740, *p* < 0.001), and this correlation was higher than that for CT (|rmax| = 0.619, *p* < 0.001). In addition, the volume of DECT‐habitat_c showed the strongest association with the area of the histopathological components (rRBCs = −0.495; rFP = 0.740) (Figure 7C). Small subgroup analysis is detailed in the Appendix E1 and Figure S3 (Supporting Information).

## 3 Discussion

Improvements in noninvasive and visual methods used to present TH during MT hold clinical value. This study aimed to develop a quantitative and visual measure of TH on multiparametric DECT scans and test its performance for predicting the stroke source and response to MT. The predictive model using intra‐thrombus diversity features showed good performance for predicting the stroke source and clinical outcomes in the external validation sets. The optimal performance for predicting the stroke source (AUC, 0.923) and clinical outcome (AUC, 0.869) was achieved using TH features in DECT. Moreover, we found that habitat_c derived from DECT scans showed the greatest positive correlation with the FP component, while habitat_b derived from CT scans showed the most correlation with the RBC component.

Previous studies used clot‐based radiomics data derived from CT images to construct models for predicting stroke source and clinical outcomes. One stroke‐source prediction model achieved an AUC of 0.838 and bridged the radiomics data and the histopathological findings for the components.^[^ ^18^ ^]^ Another model based on intra‐thrombus and peri‐thrombus regions predicted intracranial hemorrhage after MT with an AUC of 0.850.^[^ ^26^ ^]^ Despite the known associations among stroke source, clinical outcomes, and thrombotic composition,^[^ ^11^, ^27^, ^28^ ^]^ previous quantitative imaging analysis approaches could not fully establish their correspondence with TH on CT images. Clustering similar voxels across multi‐sequence images into habitats can help identify regions with similar tissue traits voxel‐by‐voxel,^[^ ^29^ ^]^ achieving success in the evaluation of various cancers.^[^ ^25^, ^30^, ^31^ ^]^ In the CT study, the performance of the radiomics models were similar to that reported in previous studies. However, when the CT‐based TH features were added to the radiomics model, the performance showed no statistical improvement.

As a new imaging technique, DECT offers new applications in studies of thrombosis in stroke patients. A previous study reported that thrombus concentrations measured on iodine‐concentration images showed the strongest association with the proportion of RBCs and FP.^[^ ^32^ ^]^ However, only a few published neuroradiology studies have used radiomics data derived from DECT images, and most of these studies have focused on whole‐tissue data.^[^ ^33^, ^34^, ^35^ ^]^ In the present study, DECT‐based models showed higher prediction performance than models based on CT, which is considered to be the optimal examination modality. In the DECT study, the TH‐related models performed better than the radiomics model for predicting stroke source and clinical outcome. Multiparametric DECT methods acquire more precise information regarding the thrombus,^[^ ^36^ ^]^ and may have an emerging role in habitat clustering. DECT‐based thrombus characterization can guide thrombectomy techniques by prompting the use of more effective devices for higher fibrin thrombi, thereby enhancing procedural success. Clinicians may choose aggressive anticoagulation, such as direct oral anticoagulants, to reduce cardioembolic stroke recurrence, especially in atrial fibrillation patients, while fibrin‐rich or cardiogenic thrombi, which are often less responsive to thrombolysis, necessitate urgent MT.^[^ ^12^ ^]^ This prioritizes patients for immediate intervention, improving outcomes by reducing reperfusion time.

Routine medical imaging can serve as a noninvasive solution for capturing TH. For example, a previous study visualized thrombus enhancement on thin‐slab maximum intensity projection computed tomography angiography scans to predict stroke sources and identify thrombi with higher FP and lower RBC content.^[^ ^37^ ^]^ However, the post‐processing protocol in this method was cumbersome, and the transferability of this method to other CT modalities was limited. Moreover, in clinical practice, the visualization of the signs depended on the experience level of the observers, limiting the wider applicability of this method. Connections between spatial radiomics features and biological markers have been reported.^[^ ^38^, ^39^, ^40^ ^]^ In the current study, a cluster method of intra‐thrombosis subregion partitioning performed well in both CT and DECT modalities. The volume of CT‐habitat_c showed the strongest positive association with the FP area in DECT scans, while the volume of DECT‐habitat_b showed the strongest positive association with the RBC area in CT scans. Although the main components of thrombi are RBCs and FP,^[^ ^41^ ^]^ the influence of other components in the thrombus may have resulted in these variations in the optimal visualization of these components. In addition, the thrombotic subregion showed stronger visualization in our study and could be quantitatively analyzed.

This study had some limitations. First, our findings underwent limited external validation, indicating the need to broaden the central values and include a wider range of data. Future work should focus on prospective validation studies with larger, more diverse cohorts to further establish the robustness and generalizability of our results. Second, despite utilizing CTP to extract CTA as an alternative to conventional CT, patients cannot undergo admission CT and DECT simultaneously, necessitating animal studies to clarify the superiority of DECT. Third, we only selected the DECT parameter maps that are commonly used in neurological disease research. Future studies should explore the impact of different combinations on the results. Finally, the pathological section of thrombus could not be accurately matched with the images in space, and the impact of components with low content inside the thrombus (such as cholesterol crystals, white blood cells) on the results needs further research.

In conclusion, the DECT‐based thrombus heterogeneity‐related models achieved the best performance for predicting stroke source and clinical outcome, and subregion analyses may help visualize thrombus components. Future research should explore MT navigation assistance and regulation of thrombolytic drug research on the basis of thrombosis component visualization by incorporating multimodal data.

## 4 Experimental Section

### Study Population

This multi‐center retrospective study was approved by the ethics review board of our institute (No. 2020‐212). This study adhered to the tenets of the Declaration of Helsinki and the Standards for Reporting of Diagnostic Accuracy Studies criteria.^[^ ^42^ ^]^

The study population consisted of consecutive patients who presented with acute ischemic stroke at four centers between June 2015 and December 2023. The inclusion criteria were as follows: 1) acute ischemic stroke caused by anterior circulation large‐vessel occlusion; 2) occlusion with a visible thrombus on admission CT/DECT scans; 3) MT performed immediately after CT/DECT scans; 4) histological analysis of the excised thrombus. The exclusion criteria for this study included: 1) patients with a history of prior MT before the CT examination; 2) thrombi that were too small or fragmented to allow for reliable histological analysis; 3) poor image or histopathology quality that precluded accurate diagnosis; and 4) patients with incomplete clinical or imaging data. A total of 255 and 221 patients were eligible for the CT and DECT studies, respectively. Among the patients eligible for the DECT study, those who underwent multimodal CT imaging (including CTP) were enrolled in the comparative set.

Basic patient information, including data regarding sex, age, smoking habits, history of hypertension, hyperlipidemia, atrial fibrillation, diabetes mellitus, coronary artery disease, admission National Institutes of Health Stroke Scale score, and occlusive site, were recorded. Two neurologists with 5 years of experience in interventional neuroradiology performed the Trial of Org 10172 in Acute Stroke Treatment (TOAST) assessments to identify cardiogenic and non‐cardiogenic thrombi on the basis of diagnostic and clinical information and obtained modified Rankin Scale scores at 90 days; scores less than 3 indicated good outcomes.

### CT Procedures and Image Evaluation

For details regarding the CT acquisition technique and reconstruction algorithm, see Table S5 and Appendix E2 (Supporting Information). Five parametric maps, including mixed images, iodine‐concentration images, and virtual monoenergetic (40 and 190 keV) and virtual non‐contrast images, were reconstructed and analyzed after the DECT examination. The arterial phase images from CTP were extracted and used as CTA. CTP arterial phase uses less contrast agent (40–50 mL) and starts scanning within 10–20 seconds after injection, resulting in lower imaging resolution (reconstructed section thickness: 5.0 mm) due to rapid dynamic scanning, whereas CTA requires more contrast agent (80–100 mL), starts scanning later (20–30 s), and provides higher resolution (reconstructed section thickness: 1.0 mm). All CT images were normalized by scaling the image intensity to 0–600 Hounsfield units (HU) and resampling all images at the same resolution (voxel size, 1 × 1 × 1 mm) before extracting the radiomics features. This normalization process partially addressed the heterogeneity of the data obtained from different centers and types of equipment; the voxel intensity values were discretized and normalized by filters that reduced image noise. The 3D thrombotic region was manually delineated by two radiologists with 5 years’ experience in neuroradiology using ITK‐SNAP (version 3.8.0; http://www.itksnap.org/pmwiki/pmwiki.php) software under the supervision of a senior radiologist with 15 years’ experience in neuroradiology.

The TH was characterized by using two and five different sequences obtained from admission CT and DECT scans, respectively. Subsequently, the K‐means module in the scikit‐learn Python package (https://scikit learn.org/stable/index.html) was used for clustering habitat subregions. The individual voxels in each cluster were grouped on the basis of their similarities by using the K‐means algorithm based on cohort, with squared Euclidean distances between voxel intensities serving as the similarity metric. All voxels were assigned to one of the clusters and visualized as spatial habitats in the original image space. The Calinski–Harabasz score, Silhouette coefficient, and Davies–Bouldin index were used to evaluate the effectiveness of different clustering configurations in selecting the best number of clusters comprehensively, with a cluster range from two to five, and each subregion was recorded as “habitat_x.” The volume and proportion of each habitat were calculated.

### Feature Selection and Model Construction

A total of 107 radiomics features (18 histogram‐based, 14 shape‐based, and 75 texture‐based) (Table S6, Supporting Information) were extracted from the whole thrombus and each subregion using PyRadiomics (version 3.1; https://pyradiomics.readthedocs.io).^[^ ^43^ ^]^ All the previous features were standardized using Z‐scores to ensure a normal distribution. The feature‐reduction methods included the *t*‐test, Pearson correlation analysis. Least absolute shrinkage and selection operator analysis with tenfold cross‐validation was used to select the most representative features. For modeling, we used random forest, logistic regression, support vector machine, k‐nearest neighbor, extra trees, light gradient boosting machine, multi‐layer perceptron, and extreme gradient boosting as classifiers. Models with high accuracy and strong generalization ability were included in the final model selection. The habitats models were constructed by early fusion of the features extracted from each habitat, and the combined model was constructed by integrating radiomics and habitats data and each habitat model through logistic regression.

### Histological Assessment

Histopathologic analyses were performed to further explore the correlation with the distribution of subregions. Whole slices of hematoxylin and eosin–stained specimens of the retrieved thrombi were photographed using image review software (Case Viewer, 3D HISTECH). The areas and proportions of RBCs and FP were analyzed manually by an experienced observer blinded to the clinical and imaging data using ImageJ software (ImageJ 1.47n; National Institutes of Health). The constituent proportions were expressed as percentage values of the respective areas.

### Statistical Analysis

Continuous variables were analyzed using Wilcoxon–Mann–Whitney and Student's *t*‐tests depending on the normality of the distribution. Categorical variables were analyzed using the χ^2^ test or Fisher's exact test. In each sequence, 107 radiomic features were extracted from each ROI and standardized using Z‐scores. Normally distributed data are presented with the mean and standard deviation. The performance of the different models was evaluated by measuring the area under the curve (AUC) of the receiver operating characteristic curve and determining the Matthews correlation coefficient. The accuracy, sensitivity, specificity, and positive and negative predictive values were also calculated. Pearson correlation coefficients were determined to evaluate the associations between the subregions and histopathologic composition. Radiomics and statistical analyses were performed using Python (version 3.7.4; Python Software Foundation) and SPSS (Version 21.0, IBM Corp.) software by an author with 10 years of experience performing statistical analysis. All statistical tests were two‐sided, and P < 0.05 was considered to indicate statistical significance.

## Conflict of Interest

The authors declare no conflict of interest.

## Supporting information



Supporting Information

## Data Availability

The data that support the findings of this study are available from the corresponding author upon reasonable request.
