# Supplementary material for: Dual‐Energy CT‐Based Assessment of Thrombotic Heterogeneity for Predicting Stroke Source and Response to Machine Thrombectomy: A Step Toward Visualization Thrombus Treatment
Source: Adv Sci (Weinh). 2025 May 28;12(32):e17295. doi: 10.1002/advs.202417295 (PMC12407335; doi:10.1002/advs.202417295)
Supplement: Supplementary file 1 — Supporting Information [file ADVS-12-e17295-s001.docx]

**Dual-energy CT-based Assessment of Thrombotic Heterogeneity for Predicting Stroke Source and Response to Machine Thrombectomy:** *A step towards visualization thrombus treatment*

*Jingxuan Jiang，Sijia Wang, Fan Xiao, Hongmei Gu**, Hao Tian, Baohui Guan, Kai Sheng，Yijia Xiong，Huilin Zhao，Minda Li，Zheng Sun，Haiyan Du，Wenxian Du*，YuehuaLi**

**
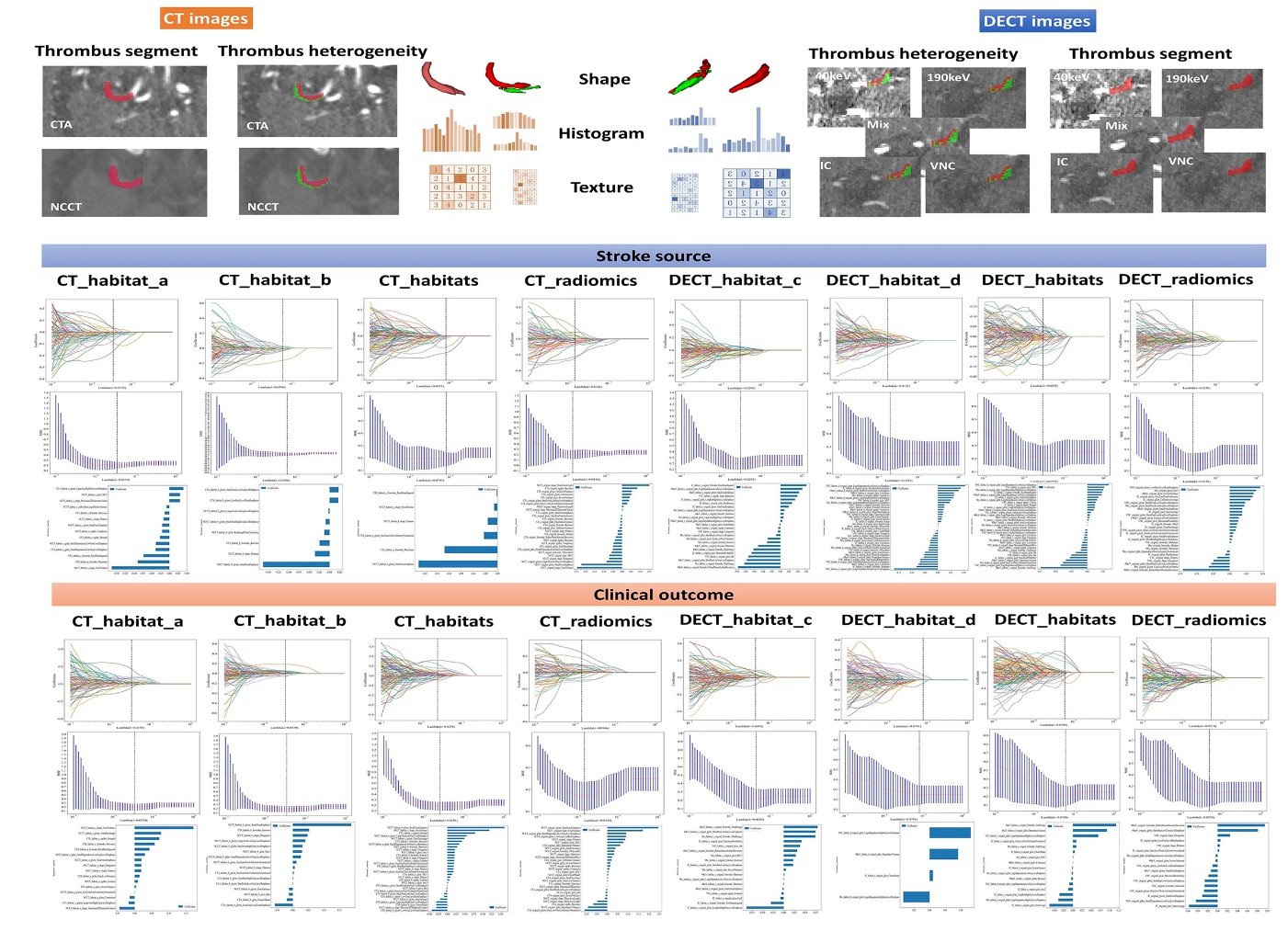
**

**Figure S1.** The LASSO regression for feature selection of the two habitats and whole thrombus of CT and DECT.


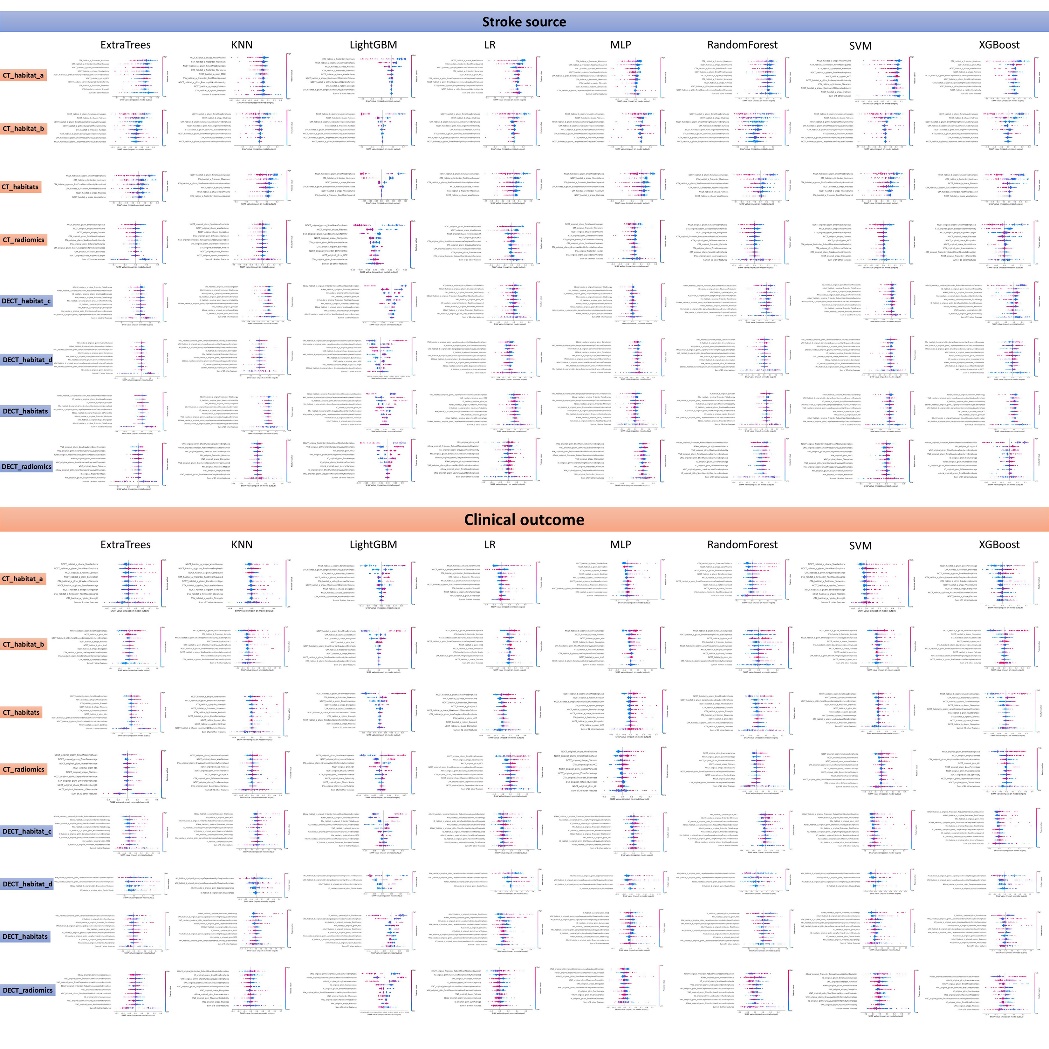


**Figure S2.** SHAP summary dot plot of different machine learning algorithms in predicting stroke source and clinical outcomes based on CT and DECT.

**Appendix E1.**

In predicting stroke source, DECT-based models outperformed CT-based models in both MCA and ICA analyses. For MCA, DECT-combined achieved the highest AUC (0.819, 95% CI = [0.714, 0.923]), while for ICA, DECT-habitats had the best performance (AUC = 0.996, 95% CI = [0.975, 1.017]). DeLong tests showed significant superiority of DECT models over CT models in multiple comparisons. In predicting clinical outcomes, DECT-based models outperformed CT-based models in both MCA and ICA analyses. For MCA, DECT-combined achieved the highest AUC (0.992, 95% CI = [0.957, 1.027]), while for ICA, DECT-habitat_c had the best performance (AUC = 0.954, 95% CI = [0.867, 1.041]). DeLong tests showed significant superiority of DECT models over CT models in multiple comparisons. In predicting stroke source, DECT-based models outperformed CT-based models in both analyses with and without atrial fibrillation. For the analysis without atrial fibrillation, DECT-habitat_c achieved the highest AUC (0.871, 95% CI = [0.786, 0.956]), while for the analysis with atrial fibrillation, DECT-habitat_c and DECT-combined had the best performance (both with AUC = 0.963, 95% CI = [0.902, 1.023]). DeLong tests showed significant superiority of DECT models over CT models in multiple comparisons. In predicting clinical outcomes, DECT-based models outperformed CT-based models in both analyses with and without atrial fibrillation. For the analysis with atrial fibrillation, DECT-habitats and DECT-combined achieved the highest AUC (both 0.990, 95% CI = [0.942, 1.039]), while DECT-habitat_c also showed strong performance (AUC = 0.918, 95% CI = [0.781, 1.055]). For the analysis without atrial fibrillation, DECT-habitats had the best performance (AUC = 0.909, 95% CI = [0.812, 1.007]), followed by DECT-combined (AUC = 0.907, 95% CI = [0.808, 1.006]) and DECT-habitat_c (AUC = 0.905, 95% CI = [0.805, 1.005]). DeLong tests showed significant superiority of DECT models over CT models in multiple comparisons **(Figure S3)**.


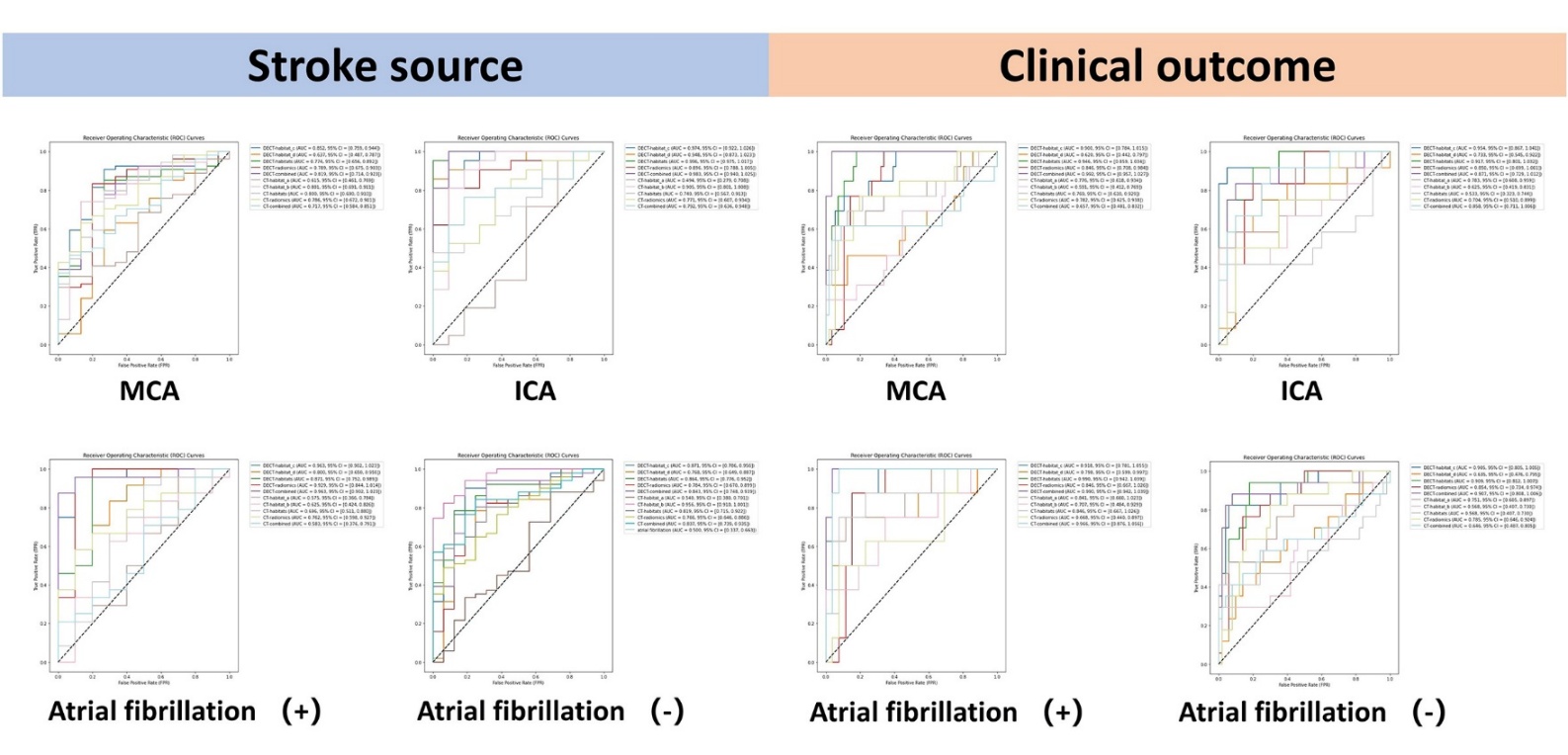


**Figure S3.** The ROC curves for predicting the stroke source and clinical outcome based on CT and DECT in different subgroups.

**Table S1:** Baseline clinical characteristics of the patients grouped by stroke source

| **Characteristics** | **CT** | | | | | | | | | **DECT** | | | | | | | | |
| --- | --- | --- | --- | --- | --- | --- | --- | --- | --- | --- | --- | --- | --- | --- | --- | --- | --- | --- |
|  | **Train** | | | **Test** | | | **Validation** | | | **Train** | | | **Test** | | | **Validation** | | |
|  | **NCE** | **CE** | **p value** | **NCE** | **CE** | **p value** | **NCE** | **CE** | **p value** | **NCE** | **CE** | **p value** | **NCE** | **CE** | **p value** | **NCE** | **CE** | **p value** |
| **Age** | 71.38±11.64 | 73.69±11.95 | 0.185 | 67.32±10.85 | 68.45±11.71 | 0.712 | 60.82±11.73 | 61.82±12.26 | 0.632 | 69.89±13.56 | 71.34±8.78 | 0.912 | 65.00±11.61 | 66.20±12.25 | 0.776 | 70.27±11.18 | 67.27±15.78 | 0.399 |
| **NIHSS** | 15.00±7.40 | 15.61±9.24 | 0.875 | 11.48±6.13 | 11.35±6.00 | 0.947 | 14.50±5.66 | 13.06±6.45 | 0.371 | 12.35±6.11 | 12.87±6.31 | 0.686 | 11.55±4.08 | 10.60±5.04 | 0.294 | 8.77±6.48 | 12.16±7.31 | 0.033 |
| **Sex** |  |  | 1 |  |  | 1 |  |  | 0.997 |  |  | 1 |  |  | 0.625 |  |  | 0.811 |
| Absent | 23(47.92) | 39(48.75) |  | 8(32.00) | 11(35.48) |  | 6(27.27) | 15(30.61) |  | 17(45.95) | 30(44.12) |  | 5(45.45) | 11(31.43) |  | 11(42.31) | 16(36.36) |  |
| present | 25(52.08) | 41(51.25) |  | 17(68.00) | 20(64.52) |  | 16(72.73) | 34(69.39) |  | 20(54.05) | 38(55.88) |  | 6(54.55) | 24(68.57) |  | 15(57.69) | 28(63.64) |  |
| **Atrial fibrillation** | |  | 0.927 |  |  | 1 |  |  | 1 |  |  | 0.05 |  |  | 0.24 |  |  | 0.982 |
| Absent | 24(50.00) | 42(52.50) |  | 17(68.00) | 22(70.97) |  | 14(63.64) | 30(61.22) |  | 20(54.05) | 22(32.35) |  | 7(63.64) | 30(85.71) |  | 18(69.23) | 29(65.91) |  |
| present | 24(50.00) | 38(47.50) |  | 8(32.00) | 9(29.03) |  | 8(36.36) | 19(38.78) |  | 17(45.95) | 46(67.65) |  | 4(36.36) | 5(14.29) |  | 8(30.77) | 15(34.09) |  |
| **Smoke** |  |  | 0.276 |  |  | 0.089 |  |  | 0.525 |  |  | 0.932 |  |  | 0.771 |  |  | 0.479 |
| Absent | 37(77.08) | 69(86.25) |  | 14(56.00) | 25(80.65) |  | 18(81.82) | 35(71.43) |  | 30(81.08) | 57(83.82) |  | 9(81.82) | 25(71.43) |  | 16(61.54) | 32(72.73) |  |
| present | 11(22.92) | 11(13.75) |  | 11(44.00) | 6(19.35) |  | 4(18.18) | 14(28.57) |  | 7(18.92) | 11(16.18) |  | 2(18.18) | 10(28.57) |  | 10(38.46) | 12(27.27) |  |
| **Hypertension** | |  | 0.918 |  |  | 0.69 |  |  | 0.731 |  |  | 0.152 |  |  | 0.066 |  |  | 0.038 |
| Absent | 12(25.00) | 22(27.50) |  | 6(24.00) | 5(16.13) |  | 6(27.27) | 17(34.69) |  | 18(48.65) | 22(32.35) |  | 1(9.09) | 16(45.71) |  | 15(57.69) | 13(29.55) |  |
| present | 36(75.00) | 58(72.50) |  | 19(76.00) | 26(83.87) |  | 16(72.73) | 32(65.31) |  | 19(51.35) | 46(67.65) |  | 10(90.91) | 19(54.29) |  | 11(42.31) | 31(70.45) |  |
| **Hyperlipidemia** | |  | 0.178 |  |  | 0.499 |  |  | 0.13 |  |  | 0.806 |  |  | 0.575 |  |  | 0.757 |
| Absent | 44(91.67) | 65(81.25) |  | 22(88.00) | 24(77.42) |  | 18(81.82) | 47(95.92) |  | 35(94.59) | 62(91.18) |  | 11(100.00) | 31(88.57) |  | 20(76.92) | 31(70.45) |  |
| present | 4(8.33) | 15(18.75) |  | 3(12.00) | 7(22.58) |  | 4(18.18) | 2(4.08) |  | 2(5.41) | 6(8.82) |  | null | 4(11.43) |  | 6(23.08) | 13(29.55) |  |
| **Diabetes** |  |  | 1 |  |  | 0.626 |  |  | 0.244 |  |  | 1 |  |  | 1 |  |  | 0.386 |
| Absent | 33(68.75) | 55(68.75) |  | 17(68.00) | 24(77.42) |  | 15(68.18) | 41(83.67) |  | 29(78.38) | 53(77.94) |  | 8(72.73) | 25(71.43) |  | 21(80.77) | 30(68.18) |  |
| present | 15(31.25) | 25(31.25) |  | 8(32.00) | 7(22.58) |  | 7(31.82) | 8(16.33) |  | 8(21.62) | 15(22.06) |  | 3(27.27) | 10(28.57) |  | 5(19.23) | 14(31.82) |  |
| **Coronary_disease** | |  | 0.91 |  |  | 0.705 |  |  | 0.439 |  |  | 0.31 |  |  | 1 |  |  | 0.395 |
| Absent | 39(81.25) | 63(78.75) |  | 22(88.00) | 25(80.65) |  | 17(77.27) | 43(87.76) |  | 36(97.30) | 61(89.71) |  | 11(100.00) | 33(94.29) |  | 22(84.62) | 32(72.73) |  |
| present | 9(18.75) | 17(21.25) |  | 3(12.00) | 6(19.35) |  | 5(22.73) | 6(12.24) |  | 1(2.70) | 7(10.29) |  | null | 2(5.71) |  | 4(15.38) | 12(27.27) |  |
| **Location** |  |  | 0.185 |  |  | 0.322 |  |  | 0.776 |  |  | 0.284 |  |  | 0.671 |  |  | 0.531 |
| MCA | 29(60.42) | 60(75.00) |  | 17(68.00) | 26(83.87) |  | 15(68.18) | 34(69.39) |  | 23(62.16) | 52(76.47) |  | 5(45.45) | 18(51.43) |  | 21(80.77) | 33(75.00) |  |
| ICA | 14(29.17) | 13(16.25) |  | 6(24.00) | 3(9.68) |  | 7(31.82) | 14(28.57) |  | 12(32.43) | 13(19.12) |  | 4(36.36) | 14(40.00) |  | 5(19.23) | 9(20.45) |  |
| MCA+ICA | 5(10.42) | 7(8.75) |  | 2(8.00) | 2(6.45) |  | null | 1(2.04) |  | 2(5.41) | 3(4.41) |  | 2(18.18) | 3(8.57) |  | null | 2(4.55) |  |

**CT=**computed tomography, **DECT=**dual-energy CT, **CE**=cardiogenic, **NCE**=non- cardiogenic, **NIHSS=**National Institutes of Health Stroke Scale, **MCA**=middle cerebral artery, **ICA**=Internal carotid artery.

**Table S2:** Baseline clinical characteristics of the patients grouped by clinical outcomes

| **Characteristics** | **CT** | | | | | | | | | **DECT** | | | | | | | | |
| --- | --- | --- | --- | --- | --- | --- | --- | --- | --- | --- | --- | --- | --- | --- | --- | --- | --- | --- |
|  | **Train** | | | **Test** | | | **Validation** | | | **Train** | | | **Test** | | | **Validation** | | |
|  | **mRS>3** | **mRS<3** | **p value** | **mRS>3** | **mRS<3** | **p value** | **mRS>3** | **mRS<3** | **p value** | **mRS>3** | **mRS<3** | **p value** | **mRS>3** | **mRS<3** | **p value** | **mRS>3** | **mRS<3** | **p value** |
| **Age** | 73.09±11.67 | 72.39±12.22 | 0.638 | 69.24±11.41 | 65.95±10.95 | 0.291 | 61.76±12.01 | 61.17±12.24 | 0.802 | 71.31±9.16 | 69.95±13.09 | 0.825 | 67.60±10.98 | 62.75±13.48 | 0.194 | 67.35±15.67 | 70.81±10.04 | 0.355 |
| **NIHSS** | 15.06±9.06 | 15.90±7.79 | 0.447 | 11.76±6.63 | 10.86±4.98 | 0.717 | 12.76±6.76 | 14.53±5.32 | 0.237 | 12.47±6.30 | 13.08±6.12 | 0.633 | 10.13±4.83 | 12.12±4.62 | 0.12 | 12.06±6.97 | 8.19±7.01 | 0.017 |
| **Sex** |  |  | 1 |  |  | 0.256 |  |  | 0.167 |  |  | 0.663 |  |  | 1 |  |  | 0.83 |
| Absent | 38(48.10) | 24(48.98) |  | 14(41.18) | 5(22.73) |  | 9(21.95) | 12(40.00) |  | 32(47.06) | 15(40.54) |  | 10(33.33) | 6(37.50) |  | 18(36.73) | 9(42.86) |  |
| present | 41(51.90) | 25(51.02) |  | 20(58.82) | 17(77.27) |  | 32(78.05) | 18(60.00) |  | 36(52.94) | 22(59.46) |  | 20(66.67) | 10(62.50) |  | 31(63.27) | 12(57.14) |  |
| **Atrial fibrillation** | |  | 1 |  |  | 0.483 |  |  | 0.15 |  |  | 0.05 |  |  | 0.064 |  |  | 0.824 |
| Absent | 41(51.90) | 25(51.02) |  | 22(64.71) | 17(77.27) |  | 22(53.66) | 22(73.33) |  | 22(32.35) | 20(54.05) |  | 27(90.00) | 10(62.50) |  | 32(65.31) | 15(71.43) |  |
| present | 38(48.10) | 24(48.98) |  | 12(35.29) | 5(22.73) |  | 19(46.34) | 8(26.67) |  | 46(67.65) | 17(45.95) |  | 3(10.00) | 6(37.50) |  | 17(34.69) | 6(28.57) |  |
| **Smoke** |  |  | 0.138 |  |  | 1 |  |  | 0.953 |  |  | 0.531 |  |  | 0.635 |  |  | 0.028 |
| Absent | 69(87.34) | 37(75.51) |  | 24(70.59) | 15(68.18) |  | 30(73.17) | 23(76.67) |  | 58(85.29) | 29(78.38) |  | 21(70.00) | 13(81.25) |  | 38(77.55) | 10(47.62) |  |
| present | 10(12.66) | 12(24.49) |  | 10(29.41) | 7(31.82) |  | 11(26.83) | 7(23.33) |  | 10(14.71) | 8(21.62) |  | 9(30.00) | 3(18.75) |  | 11(22.45) | 11(52.38) |  |
| **Hypertension** | |  | 0.541 |  |  | 0.572 |  |  | 0.36 |  |  | 0.152 |  |  | 0.122 |  |  | 0.558 |
| Absent | 19(24.05) | 15(30.61) |  | 8(23.53) | 3(13.64) |  | 11(26.83) | 12(40.00) |  | 22(32.35) | 18(48.65) |  | 14(46.67) | 3(18.75) |  | 18(36.73) | 10(47.62) |  |
| present | 60(75.95) | 34(69.39) |  | 26(76.47) | 19(86.36) |  | 30(73.17) | 18(60.00) |  | 46(67.65) | 19(51.35) |  | 16(53.33) | 13(81.25) |  | 31(63.27) | 11(52.38) |  |
| **Hyperlipidemia** | |  | 0.054 |  |  | 0.262 |  |  | 0.01 |  |  | 1 |  |  | 0.327 |  |  | 1 |
| Absent | 63(79.75) | 46(93.88) |  | 30(88.24) | 16(72.73) |  | 41(100.00) | 24(80.00) |  | 63(92.65) | 34(91.89) |  | 26(86.67) | 16(100.00) | | 36(73.47) | 15(71.43) |  |
| present | 16(20.25) | 3(6.12) |  | 4(11.76) | 6(27.27) |  | null | 6(20.00) |  | 5(7.35) | 3(8.11) |  | 4(13.33) | null |  | 13(26.53) | 6(28.57) |  |
| **Diabetes** |  |  | 0.477 |  |  | 0.808 |  |  | 0.203 |  |  | 1 |  |  | 0.988 |  |  | 1 |
| Absent | 52(65.82) | 36(73.47) |  | 24(70.59) | 17(77.27) |  | 35(85.37) | 21(70.00) |  | 53(77.94) | 29(78.38) |  | 21(70.00) | 12(75.00) |  | 36(73.47) | 15(71.43) |  |
| present | 27(34.18) | 13(26.53) |  | 10(29.41) | 5(22.73) |  | 6(14.63) | 9(30.00) |  | 15(22.06) | 8(21.62) |  | 9(30.00) | 4(25.00) |  | 13(26.53) | 6(28.57) |  |
| **Coronary_disease** | |  | 1 |  |  | 0.44 |  |  | 0.219 |  |  | 0.806 |  |  | 0.766 |  |  | 1 |
| Absent | 63(79.75) | 39(79.59) |  | 27(79.41) | 20(90.91) |  | 37(90.24) | 23(76.67) |  | 62(91.18) | 35(94.59) |  | 28(93.33) | 16(100.00) | | 38(77.55) | 16(76.19) |  |
| present | 16(20.25) | 10(20.41) |  | 7(20.59) | 2(9.09) |  | 4(9.76) | 7(23.33) |  | 6(8.82) | 2(5.41) |  | 2(6.67) | null |  | 11(22.45) | 5(23.81) |  |
| **Location** |  |  | 0.112 |  |  | 0.147 |  |  | 0.6 |  |  | 0.539 |  |  | 0.962 |  |  | 0.352 |
| MCA | 60(75.95) | 29(59.18) |  | 29(85.29) | 14(63.64) |  | 29(70.73) | 20(66.67) |  | 51(75.00) | 24(64.86) |  | 15(50.00) | 8(50.00) |  | 39(79.59) | 15(71.43) |  |
| ICA | 14(17.72) | 13(26.53) |  | 3(8.82) | 6(27.27) |  | 11(26.83) | 10(33.33) |  | 14(20.59) | 11(29.73) |  | 12(40.00) | 6(37.50) |  | 8(16.33) | 6(28.57) |  |
| MCA+ICA | 5(6.33) | 7(14.29) |  | 2(5.88) | 2(9.09) |  | 1(2.44) | null |  | 3(4.41) | 2(5.41) |  | 3(10.00) | 2(12.50) |  | 2(4.08) | null |  |

**CT=**computed tomography, **DECT=**dual-energy CT, mRS=modified Rankin Scale, **NIHSS=**National Institutes of Health Stroke Scale, **MCA**=middle cerebral artery, **ICA**=Internal carotid artery.

**Table S3:** The performance of different classifiers in predicting stroke source

| **Classifiers** | **Cohorts** | **Accuracy** | **AUC** | **95% CI** | **Sensitivity** | **Specificity** | **PPV** | **NPV** | **Precision** | **Recall** | **F1** | **Threshold** |
| --- | --- | --- | --- | --- | --- | --- | --- | --- | --- | --- | --- | --- |
| **ExtraTrees** | CT_habitat_a-test | 0.607 | 0.59 | 0.4347 - 0.7459 | 0.742 | 0.44 | 0.622 | 0.579 | 0.622 | 0.742 | 0.676 | 0.5 |
|  | CT_habitat_a-train | 0.375 | 1 | 1.0000 - 1.0000 | 0 | 1 | 0 | 0.375 | 0 | 0 | NaN | 1 |
|  | CT_habitat_a-val | 0.676 | 0.699 | 0.5613 - 0.8376 | 0.653 | 0.727 | 0.842 | 0.485 | 0.842 | 0.653 | 0.736 | 0.6 |
|  | CT_habitat_b-test | 0.661 | 0.778 | 0.6557 - 0.9004 | 0.677 | 0.64 | 0.7 | 0.615 | 0.7 | 0.677 | 0.689 | 0.5 |
|  | CT_habitat_b-train | 0.375 | 1 | 1.0000 - 1.0000 | 0 | 1 | 0 | 0.375 | 0 | 0 | NaN | 1 |
|  | CT_habitat_b-val | 0.746 | 0.722 | 0.5836 - 0.8608 | 0.796 | 0.636 | 0.83 | 0.583 | 0.83 | 0.796 | 0.812 | 0.4 |
|  | CT_habitats-test | 0.714 | 0.754 | 0.6233 - 0.8838 | 0.613 | 0.84 | 0.826 | 0.636 | 0.826 | 0.613 | 0.704 | 0.8 |
|  | CT_habitats-train | 0.375 | 1 | 1.0000 - 1.0000 | 0 | 1 | 0 | 0.375 | 0 | 0 | NaN | 1 |
|  | CT_habitats-val | 0.718 | 0.708 | 0.5669 - 0.8496 | 0.816 | 0.5 | 0.784 | 0.55 | 0.784 | 0.816 | 0.8 | 0.5 |
|  | CT_radiomics-train | 0.375 | 1 | 1.0000 - 1.0000 | 0 | 1 | 0 | 0.375 | 0 | 0 | NaN | 1 |
|  | CT_radiomics-test | 0.589 | 0.765 | 0.6399 - 0.8905 | 0.258 | 1 | 1 | 0.521 | 1 | 0.258 | 0.41 | 0.8 |
|  | DECT_habitat_c-train | 0.352 | 1 | 1.0000 - 1.0000 | 0 | 1 | 0 | 0.352 | 0 | 0 | NaN | 1 |
|  | DECT_habitat_c-test | 0.674 | 0.812 | 0.6865 - 0.9369 | 0.6 | 0.909 | 0.955 | 0.417 | 0.955 | 0.6 | 0.737 | 0.6 |
|  | DECT_habitat_c-val | 0.529 | 0.778 | 0.6713 - 0.8856 | 0.273 | 0.962 | 0.923 | 0.439 | 0.923 | 0.273 | 0.421 | 0.8 |
|  | DECT_habitat_d-train | 0.352 | 1 | 1.0000 - 1.0000 | 0 | 1 | 0 | 0.352 | 0 | 0 | NaN | 1 |
|  | DECT_habitat_d-test | 0.37 | 0.744 | 0.5971 - 0.8912 | 0.171 | 1 | 1 | 0.275 | 1 | 0.171 | 0.293 | 0.8 |
|  | DECT_habitat_d-val | 0.643 | 0.614 | 0.4614 - 0.7667 | 0.659 | 0.615 | 0.744 | 0.516 | 0.744 | 0.659 | 0.699 | 0.6 |
|  | DECT_habitats-train | 0.352 | 1 | 1.0000 - 1.0000 | 0 | 1 | 0 | 0.352 | 0 | 0 | NaN | 1 |
|  | DECT_habitats-test | 0.609 | 0.79 | 0.6420 - 0.9372 | 0.514 | 0.909 | 0.947 | 0.37 | 0.947 | 0.514 | 0.667 | 0.7 |
|  | DECT_habitats-val | 0.5 | 0.675 | 0.5481 - 0.8015 | 0.25 | 0.923 | 0.846 | 0.421 | 0.846 | 0.25 | 0.386 | 0.8 |
|  | DECT_radiomics-train | 0.352 | 1 | 1.0000 - 1.0000 | 0 | 1 | 0 | 0.352 | 0 | 0 | NaN | 1 |
|  | DECT_radiomics-test | 0.543 | 0.803 | 0.6757 - 0.9295 | 0.4 | 1 | 1 | 0.344 | 1 | 0.4 | 0.571 | 0.7 |
|  | DECT_radiomics-val | 0.629 | 0.666 | 0.5368 - 0.7954 | 0.727 | 0.462 | 0.696 | 0.5 | 0.696 | 0.727 | 0.711 | 0.5 |
| **KNN** | CT_radiomics-val | 0.549 | 0.725 | 0.5979 - 0.8520 | 0.429 | 0.818 | 0.84 | 0.391 | 0.84 | 0.429 | 0.568 | 0.7 |
|  | CT_habitat_a-test | 0.518 | 0.546 | 0.3969 - 0.6960 | 0.774 | 0.2 | 0.545 | 0.417 | 0.545 | 0.774 | 0.64 | 0.4 |
|  | CT_habitat_a-train | 0.773 | 0.929 | 0.8882 - 0.9693 | 0.637 | 1 | 1 | 0.623 | 1 | 0.637 | 0.779 | 0.8 |
|  | CT_habitat_a-val | 0.606 | 0.628 | 0.4878 - 0.7682 | 0.673 | 0.455 | 0.733 | 0.385 | 0.733 | 0.673 | 0.702 | 0.6 |
|  | CT_habitat_b-test | 0.607 | 0.821 | 0.7125 - 0.9287 | 0.323 | 0.96 | 0.909 | 0.533 | 0.909 | 0.323 | 0.476 | 0.8 |
|  | CT_habitat_b-train | 0.797 | 0.908 | 0.8595 - 0.9556 | 0.737 | 0.896 | 0.922 | 0.672 | 0.922 | 0.737 | 0.819 | 0.6 |
|  | CT_habitat_b-val | 0.789 | 0.834 | 0.7188 - 0.9491 | 0.816 | 0.727 | 0.87 | 0.64 | 0.87 | 0.816 | 0.842 | 0.4 |
|  | CT_habitats-test | 0.696 | 0.809 | 0.6977 - 0.9203 | 0.452 | 1 | 1 | 0.595 | 1 | 0.452 | 0.622 | 0.8 |
|  | CT_habitats-train | 0.719 | 0.937 | 0.8995 - 0.9742 | 0.55 | 1 | 1 | 0.571 | 1 | 0.55 | 0.71 | 0.8 |
|  | CT_habitats-val | 0.563 | 0.799 | 0.6871 - 0.9103 | 0.408 | 0.909 | 0.909 | 0.408 | 0.909 | 0.408 | 0.563 | 0.8 |
|  | CT_radiomics-train | 0.609 | 0.908 | 0.8607 - 0.9560 | 0.375 | 1 | 1 | 0.49 | 1 | 0.375 | 0.545 | 0.8 |
|  | CT_radiomics-test | 0.643 | 0.749 | 0.6216 - 0.8765 | 0.452 | 0.88 | 0.824 | 0.564 | 0.824 | 0.452 | 0.583 | 0.8 |
|  | DECT_habitat_c-train | 0.676 | 0.912 | 0.8619 - 0.9616 | 0.5 | 1 | 1 | 0.521 | 1 | 0.5 | 0.667 | 0.8 |
|  | DECT_habitat_c-test | 0.63 | 0.883 | 0.7901 - 0.9761 | 0.514 | 1 | 1 | 0.393 | 1 | 0.514 | 0.679 | 0.8 |
|  | DECT_habitat_c-val | 0.714 | 0.819 | 0.7179 - 0.9193 | 0.705 | 0.731 | 0.816 | 0.594 | 0.816 | 0.705 | 0.756 | 0.6 |
|  | DECT_habitat_d-train | 0.524 | 0.848 | 0.7782 - 0.9178 | 0.265 | 1 | 1 | 0.425 | 1 | 0.265 | 0.419 | 0.8 |
|  | DECT_habitat_d-test | 0.283 | 0.531 | 0.3289 - 0.7334 | 0.114 | 0.818 | 0.667 | 0.225 | 0.667 | 0.114 | 0.195 | 0.8 |
|  | DECT_habitat_d-val | 0.514 | 0.519 | 0.3788 - 0.6597 | 0.523 | 0.5 | 0.639 | 0.382 | 0.639 | 0.523 | 0.575 | 0.6 |
|  | DECT_habitats-train | 0.667 | 0.915 | 0.8656 - 0.9643 | 0.485 | 1 | 1 | 0.514 | 1 | 0.485 | 0.653 | 0.8 |
|  | DECT_habitats-test | 0.543 | 0.86 | 0.7458 - 0.9736 | 0.4 | 1 | 1 | 0.344 | 1 | 0.4 | 0.571 | 0.8 |
|  | DECT_habitats-val | 0.514 | 0.726 | 0.6027 - 0.8501 | 0.295 | 0.885 | 0.812 | 0.426 | 0.812 | 0.295 | 0.433 | 0.8 |
|  | DECT_radiomics-train | 0.581 | 0.867 | 0.8015 - 0.9334 | 0.353 | 1 | 1 | 0.457 | 1 | 0.353 | 0.522 | 0.8 |
|  | DECT_radiomics-test | 0.435 | 0.778 | 0.6393 - 0.9166 | 0.257 | 1 | 1 | 0.297 | 1 | 0.257 | 0.409 | 0.8 |
|  | DECT_radiomics-val | 0.429 | 0.585 | 0.4446 - 0.7250 | 0.205 | 0.808 | 0.643 | 0.375 | 0.643 | 0.205 | 0.31 | 0.8 |
| **LightGBM** | CT_radiomics-val | 0.563 | 0.704 | 0.5732 - 0.8349 | 0.449 | 0.818 | 0.846 | 0.4 | 0.846 | 0.449 | 0.587 | 0.8 |
|  | CT_habitat_a-test | 0.643 | 0.622 | 0.4707 - 0.7732 | 0.677 | 0.6 | 0.677 | 0.6 | 0.677 | 0.677 | 0.677 | 0.62 |
|  | CT_habitat_a-train | 0.844 | 0.926 | 0.8819 - 0.9692 | 0.837 | 0.854 | 0.905 | 0.759 | 0.905 | 0.837 | 0.87 | 0.615 |
|  | CT_habitat_a-val | 0.676 | 0.589 | 0.4329 - 0.7443 | 0.755 | 0.5 | 0.771 | 0.478 | 0.771 | 0.755 | 0.763 | 0.603 |
|  | CT_habitat_b-test | 0.75 | 0.847 | 0.7454 - 0.9488 | 0.839 | 0.64 | 0.743 | 0.762 | 0.743 | 0.839 | 0.788 | 0.592 |
|  | CT_habitat_b-train | 0.828 | 0.917 | 0.8690 - 0.9643 | 0.8 | 0.875 | 0.914 | 0.724 | 0.914 | 0.8 | 0.853 | 0.592 |
|  | CT_habitat_b-val | 0.662 | 0.759 | 0.6265 - 0.8920 | 0.592 | 0.818 | 0.879 | 0.474 | 0.879 | 0.592 | 0.707 | 0.665 |
|  | CT_habitats-test | 0.75 | 0.8 | 0.6799 - 0.9201 | 0.645 | 0.88 | 0.87 | 0.667 | 0.87 | 0.645 | 0.741 | 0.671 |
|  | CT_habitats-train | 0.852 | 0.94 | 0.9020 - 0.9774 | 0.85 | 0.854 | 0.907 | 0.774 | 0.907 | 0.85 | 0.877 | 0.561 |
|  | CT_habitats-val | 0.803 | 0.784 | 0.6598 - 0.9079 | 0.898 | 0.591 | 0.83 | 0.722 | 0.83 | 0.898 | 0.863 | 0.552 |
|  | CT_radiomics-train | 0.883 | 0.955 | 0.9215 - 0.9891 | 0.862 | 0.917 | 0.945 | 0.8 | 0.945 | 0.862 | 0.902 | 0.624 |
|  | CT_radiomics-test | 0.732 | 0.758 | 0.6292 - 0.8869 | 0.581 | 0.92 | 0.9 | 0.639 | 0.9 | 0.581 | 0.706 | 0.717 |
|  | DECT_habitat_c-train | 0.867 | 0.921 | 0.8721 - 0.9705 | 0.868 | 0.865 | 0.922 | 0.78 | 0.922 | 0.868 | 0.894 | 0.615 |
|  | DECT_habitat_c-test | 0.63 | 0.738 | 0.5846 - 0.8908 | 0.543 | 0.909 | 0.95 | 0.385 | 0.95 | 0.543 | 0.691 | 0.722 |
|  | DECT_habitat_c-val | 0.6 | 0.624 | 0.4933 - 0.7541 | 0.477 | 0.808 | 0.808 | 0.477 | 0.808 | 0.477 | 0.6 | 0.706 |
|  | DECT_habitat_d-train | 0.905 | 0.952 | 0.9117 - 0.9914 | 0.941 | 0.838 | 0.914 | 0.886 | 0.914 | 0.941 | 0.928 | 0.607 |
|  | DECT_habitat_d-test | 0.478 | 0.449 | 0.2616 - 0.6371 | 0.429 | 0.636 | 0.789 | 0.259 | 0.789 | 0.429 | 0.556 | 0.685 |
|  | DECT_habitat_d-val | 0.629 | 0.407 | 0.2688 - 0.5459 | 0.977 | 0.038 | 0.632 | 0.5 | 0.632 | 0.977 | 0.768 | 0.399 |
|  | DECT_habitats-train | 0.924 | 0.971 | 0.9423 - 0.9993 | 0.941 | 0.892 | 0.941 | 0.892 | 0.941 | 0.941 | 0.941 | 0.621 |
|  | DECT_habitats-test | 0.652 | 0.74 | 0.5796 - 0.9010 | 0.6 | 0.818 | 0.913 | 0.391 | 0.913 | 0.6 | 0.724 | 0.69 |
|  | DECT_habitats-val | 0.557 | 0.578 | 0.4389 - 0.7167 | 0.477 | 0.692 | 0.724 | 0.439 | 0.724 | 0.477 | 0.575 | 0.676 |
|  | DECT_radiomics-train | 0.924 | 0.968 | 0.9401 - 0.9968 | 0.882 | 1 | 1 | 0.822 | 1 | 0.882 | 0.937 | 0.665 |
|  | DECT_radiomics-test | 0.848 | 0.858 | 0.7245 - 0.9924 | 0.886 | 0.727 | 0.912 | 0.667 | 0.912 | 0.886 | 0.899 | 0.66 |
|  | DECT_radiomics-val | 0.543 | 0.567 | 0.4279 - 0.7059 | 0.477 | 0.654 | 0.7 | 0.425 | 0.7 | 0.477 | 0.568 | 0.751 |
| **LR** | DECT_habitat_c-train | 0.924 | 0.964 | 0.9271 - 1.0000 | 0.941 | 0.892 | 0.941 | 0.892 | 0.941 | 0.941 | 0.941 | 0.535 |
|  | DECT_habitat_c-test | 0.826 | 0.93 | 0.8590 - 1.0000 | 0.771 | 1 | 1 | 0.579 | 1 | 0.771 | 0.871 | 0.762 |
|  | DECT_habitat_c-val | 0.9 | 0.923 | 0.8530 - 0.9931 | 0.977 | 0.769 | 0.878 | 0.952 | 0.878 | 0.977 | 0.925 | 0.509 |
|  | DECT_habitat_d-train | 0.933 | 0.988 | 0.9735 - 1.0000 | 0.897 | 1 | 1 | 0.841 | 1 | 0.897 | 0.946 | 0.692 |
|  | DECT_habitat_d-test | 0.804 | 0.816 | 0.6351 - 0.9961 | 0.8 | 0.818 | 0.933 | 0.562 | 0.933 | 0.8 | 0.862 | 0.425 |
|  | DECT_habitat_d-val | 0.7 | 0.663 | 0.5219 - 0.8032 | 0.841 | 0.462 | 0.725 | 0.632 | 0.725 | 0.841 | 0.779 | 0.23 |
|  | DECT_habitats-train | 0.962 | 0.993 | 0.9831 - 1.0000 | 0.971 | 0.946 | 0.971 | 0.946 | 0.971 | 0.971 | 0.971 | 0.606 |
|  | DECT_habitats-test | 0.826 | 0.901 | 0.8058 - 0.9968 | 0.8 | 0.909 | 0.966 | 0.588 | 0.966 | 0.8 | 0.875 | 0.437 |
|  | DECT_habitats-val | 0.743 | 0.764 | 0.6495 - 0.8785 | 0.75 | 0.731 | 0.825 | 0.633 | 0.825 | 0.75 | 0.786 | 0.683 |
|  | DECT_radiomics-train | 0.971 | 0.986 | 0.9643 - 1.0000 | 0.985 | 0.946 | 0.971 | 0.972 | 0.971 | 0.985 | 0.978 | 0.59 |
|  | DECT_radiomics-test | 0.761 | 0.852 | 0.7180 - 0.9859 | 0.714 | 0.909 | 0.962 | 0.5 | 0.962 | 0.714 | 0.82 | 0.649 |
|  | DECT_radiomics-val | 0.743 | 0.74 | 0.6088 - 0.8719 | 0.75 | 0.731 | 0.825 | 0.633 | 0.825 | 0.75 | 0.786 | 0.633 |
|  | CT_radiomics-val | 0.62 | 0.78 | 0.6660 - 0.8943 | 0.469 | 0.955 | 0.958 | 0.447 | 0.958 | 0.469 | 0.63 | 0.762 |
|  | CT_habitat_a-test | 0.696 | 0.743 | 0.6122 - 0.8742 | 0.645 | 0.76 | 0.769 | 0.633 | 0.769 | 0.645 | 0.702 | 0.704 |
|  | CT_habitat_a-train | 0.867 | 0.904 | 0.8457 - 0.9621 | 0.912 | 0.792 | 0.88 | 0.844 | 0.88 | 0.912 | 0.896 | 0.585 |
|  | CT_habitat_a-val | 0.634 | 0.68 | 0.5415 - 0.8184 | 0.592 | 0.727 | 0.829 | 0.444 | 0.829 | 0.592 | 0.69 | 0.663 |
|  | CT_habitat_b-test | 0.804 | 0.867 | 0.7722 - 0.9619 | 0.871 | 0.72 | 0.794 | 0.818 | 0.794 | 0.871 | 0.831 | 0.607 |
|  | CT_habitat_b-train | 0.812 | 0.874 | 0.8066 - 0.9424 | 0.775 | 0.875 | 0.912 | 0.7 | 0.912 | 0.775 | 0.838 | 0.663 |
|  | CT_habitat_b-val | 0.887 | 0.881 | 0.7873 - 0.9752 | 0.898 | 0.864 | 0.936 | 0.792 | 0.936 | 0.898 | 0.917 | 0.506 |
|  | CT_habitats-test | 0.821 | 0.854 | 0.7454 - 0.9630 | 0.71 | 0.96 | 0.957 | 0.727 | 0.957 | 0.71 | 0.815 | 0.728 |
|  | CT_habitats-train | 0.828 | 0.899 | 0.8440 - 0.9534 | 0.837 | 0.812 | 0.882 | 0.75 | 0.882 | 0.837 | 0.859 | 0.595 |
|  | CT_habitats-val | 0.761 | 0.848 | 0.7496 - 0.9462 | 0.714 | 0.864 | 0.921 | 0.576 | 0.921 | 0.714 | 0.805 | 0.682 |
|  | CT_radiomics-train | 0.93 | 0.957 | 0.9197 - 0.9949 | 0.95 | 0.896 | 0.938 | 0.915 | 0.938 | 0.95 | 0.944 | 0.573 |
|  | CT_radiomics-test | 0.786 | 0.804 | 0.6869 - 0.9208 | 0.645 | 0.96 | 0.952 | 0.686 | 0.952 | 0.645 | 0.769 | 0.819 |
|  | CT_radiomics-val | 0.69 | 0.789 | 0.6824 - 0.8965 | 0.612 | 0.864 | 0.909 | 0.5 | 0.909 | 0.612 | 0.732 | 0.866 |
| **MLP** | DECT_habitat_c-train | 0.857 | 0.922 | 0.8676 - 0.9774 | 0.868 | 0.838 | 0.908 | 0.775 | 0.908 | 0.868 | 0.887 | 0.61 |
|  | DECT_habitat_c-test | 0.804 | 0.868 | 0.7416 - 0.9934 | 0.8 | 0.818 | 0.933 | 0.562 | 0.933 | 0.8 | 0.862 | 0.571 |
|  | DECT_habitat_c-val | 0.757 | 0.776 | 0.6648 - 0.8876 | 0.886 | 0.538 | 0.765 | 0.737 | 0.765 | 0.886 | 0.821 | 0.562 |
|  | DECT_habitat_d-train | 0.895 | 0.942 | 0.8962 - 0.9885 | 0.912 | 0.865 | 0.925 | 0.842 | 0.925 | 0.912 | 0.919 | 0.619 |
|  | DECT_habitat_d-test | 0.717 | 0.561 | 0.3386 - 0.7835 | 0.829 | 0.364 | 0.806 | 0.4 | 0.806 | 0.829 | 0.817 | 0.568 |
|  | DECT_habitat_d-val | 0.643 | 0.572 | 0.4245 - 0.7188 | 0.75 | 0.462 | 0.702 | 0.522 | 0.702 | 0.75 | 0.725 | 0.588 |
|  | DECT_habitats-train | 0.943 | 0.978 | 0.9526 - 1.0000 | 0.985 | 0.865 | 0.931 | 0.97 | 0.931 | 0.985 | 0.957 | 0.503 |
|  | DECT_habitats-test | 0.717 | 0.745 | 0.5903 - 0.9006 | 0.743 | 0.636 | 0.867 | 0.437 | 0.867 | 0.743 | 0.8 | 0.532 |
|  | DECT_habitats-val | 0.7 | 0.701 | 0.5750 - 0.8271 | 0.795 | 0.538 | 0.745 | 0.609 | 0.745 | 0.795 | 0.769 | 0.592 |
|  | DECT_radiomics-train | 0.933 | 0.975 | 0.9457 - 1.0000 | 0.941 | 0.919 | 0.955 | 0.895 | 0.955 | 0.941 | 0.948 | 0.66 |
|  | DECT_radiomics-test | 0.826 | 0.868 | 0.7544 - 0.9807 | 0.8 | 0.909 | 0.966 | 0.588 | 0.966 | 0.8 | 0.875 | 0.584 |
|  | DECT_radiomics-val | 0.7 | 0.71 | 0.5812 - 0.8384 | 0.727 | 0.654 | 0.78 | 0.586 | 0.78 | 0.727 | 0.753 | 0.641 |
|  | CT_habitat_a-test | 0.643 | 0.641 | 0.4935 - 0.7891 | 0.71 | 0.56 | 0.667 | 0.609 | 0.667 | 0.71 | 0.687 | 0.63 |
|  | CT_habitat_a-train | 0.852 | 0.904 | 0.8464 - 0.9625 | 0.837 | 0.875 | 0.918 | 0.764 | 0.918 | 0.837 | 0.876 | 0.619 |
|  | CT_habitat_a-val | 0.704 | 0.71 | 0.5791 - 0.8402 | 0.714 | 0.682 | 0.833 | 0.517 | 0.833 | 0.714 | 0.769 | 0.602 |
|  | CT_habitat_b-test | 0.893 | 0.938 | 0.8719 - 1.0000 | 0.839 | 0.96 | 0.963 | 0.828 | 0.963 | 0.839 | 0.897 | 0.585 |
|  | CT_habitat_b-train | 0.836 | 0.896 | 0.8371 - 0.9556 | 0.837 | 0.833 | 0.893 | 0.755 | 0.893 | 0.837 | 0.865 | 0.585 |
|  | CT_habitat_b-val | 0.789 | 0.87 | 0.7771 - 0.9631 | 0.735 | 0.909 | 0.947 | 0.606 | 0.947 | 0.735 | 0.828 | 0.594 |
|  | CT_habitats-test | 0.839 | 0.866 | 0.7582 - 0.9734 | 0.774 | 0.92 | 0.923 | 0.767 | 0.923 | 0.774 | 0.842 | 0.642 |
|  | CT_habitats-train | 0.836 | 0.899 | 0.8449 - 0.9540 | 0.85 | 0.812 | 0.883 | 0.765 | 0.883 | 0.85 | 0.866 | 0.573 |
|  | CT_habitats-val | 0.718 | 0.767 | 0.6508 - 0.8835 | 0.714 | 0.727 | 0.854 | 0.533 | 0.854 | 0.714 | 0.778 | 0.624 |
|  | CT_radiomics-train | 0.852 | 0.904 | 0.8449 - 0.9640 | 0.812 | 0.917 | 0.942 | 0.746 | 0.942 | 0.812 | 0.872 | 0.628 |
|  | CT_radiomics-test | 0.768 | 0.836 | 0.7309 - 0.9413 | 0.774 | 0.76 | 0.8 | 0.731 | 0.8 | 0.774 | 0.787 | 0.636 |
|  | CT_radiomics-val | 0.789 | 0.792 | 0.6739 - 0.9106 | 0.837 | 0.682 | 0.854 | 0.652 | 0.854 | 0.837 | 0.845 | 0.547 |
| **RandomForest** | CT_habitat_a-test | 0.607 | 0.644 | 0.4975 - 0.7903 | 0.677 | 0.52 | 0.636 | 0.565 | 0.636 | 0.677 | 0.656 | 0.5 |
|  | CT_habitat_a-train | 0.984 | 1 | 1.0000 - 1.0000 | 0.975 | 1 | 1 | 0.96 | 1 | 0.975 | 0.987 | 0.6 |
|  | CT_habitat_a-val | 0.577 | 0.576 | 0.4240 - 0.7281 | 0.653 | 0.409 | 0.711 | 0.346 | 0.711 | 0.653 | 0.681 | 0.5 |
|  | CT_habitat_b-test | 0.589 | 0.801 | 0.6860 - 0.9166 | 0.258 | 1 | 1 | 0.521 | 1 | 0.258 | 0.41 | 0.8 |
|  | CT_habitat_b-train | 0.961 | 0.999 | 0.9983 - 1.0000 | 0.937 | 1 | 1 | 0.906 | 1 | 0.937 | 0.968 | 0.6 |
|  | CT_habitat_b-val | 0.746 | 0.726 | 0.5837 - 0.8680 | 0.857 | 0.5 | 0.792 | 0.611 | 0.792 | 0.857 | 0.824 | 0.4 |
|  | CT_habitats-test | 0.696 | 0.759 | 0.6315 - 0.8859 | 0.613 | 0.8 | 0.792 | 0.625 | 0.792 | 0.613 | 0.691 | 0.7 |
|  | CT_habitats-train | 0.977 | 1 | 0.9995 - 1.0000 | 0.962 | 1 | 1 | 0.941 | 1 | 0.962 | 0.981 | 0.6 |
|  | CT_habitats-val | 0.563 | 0.663 | 0.5232 - 0.8034 | 0.49 | 0.727 | 0.8 | 0.39 | 0.8 | 0.49 | 0.608 | 0.7 |
|  | CT_radiomics-train | 0.984 | 1 | 1.0000 - 1.0000 | 0.975 | 1 | 1 | 0.96 | 1 | 0.975 | 0.987 | 0.5 |
|  | CT_radiomics-test | 0.679 | 0.786 | 0.6679 - 0.9050 | 0.548 | 0.84 | 0.81 | 0.6 | 0.81 | 0.548 | 0.654 | 0.7 |
|  | DECT_habitat_c-train | 0.952 | 0.999 | 0.9975 - 1.0000 | 0.926 | 1 | 1 | 0.881 | 1 | 0.926 | 0.962 | 0.6 |
|  | DECT_habitat_c-test | 0.804 | 0.909 | 0.8104 - 1.0000 | 0.8 | 0.818 | 0.933 | 0.562 | 0.933 | 0.8 | 0.862 | 0.5 |
|  | DECT_habitat_c-val | 0.557 | 0.723 | 0.5970 - 0.8488 | 0.386 | 0.846 | 0.81 | 0.449 | 0.81 | 0.386 | 0.523 | 0.8 |
|  | DECT_habitat_d-train | 0.971 | 1 | 0.9992 - 1.0000 | 0.956 | 1 | 1 | 0.925 | 1 | 0.956 | 0.977 | 0.6 |
|  | DECT_habitat_d-test | 0.478 | 0.668 | 0.4591 - 0.8760 | 0.4 | 0.727 | 0.824 | 0.276 | 0.824 | 0.4 | 0.538 | 0.7 |
|  | DECT_habitat_d-val | 0.457 | 0.476 | 0.3387 - 0.6132 | 0.364 | 0.615 | 0.615 | 0.364 | 0.615 | 0.364 | 0.457 | 0.7 |
|  | DECT_habitats-train | 0.962 | 0.997 | 0.9925 - 1.0000 | 0.956 | 0.973 | 0.985 | 0.923 | 0.985 | 0.956 | 0.97 | 0.6 |
|  | DECT_habitats-test | 0.717 | 0.783 | 0.6231 - 0.9431 | 0.771 | 0.545 | 0.844 | 0.429 | 0.844 | 0.771 | 0.806 | 0.5 |
|  | DECT_habitats-val | 0.529 | 0.678 | 0.5552 - 0.8006 | 0.295 | 0.923 | 0.867 | 0.436 | 0.867 | 0.295 | 0.441 | 0.8 |
|  | DECT_radiomics-train | 0.962 | 1 | 0.9992 - 1.0000 | 0.941 | 1 | 1 | 0.902 | 1 | 0.941 | 0.97 | 0.6 |
|  | DECT_radiomics-test | 0.739 | 0.883 | 0.7805 - 0.9858 | 0.686 | 0.909 | 0.96 | 0.476 | 0.96 | 0.686 | 0.8 | 0.6 |
|  | DECT_radiomics-val | 0.471 | 0.669 | 0.5358 - 0.8025 | 0.25 | 0.846 | 0.733 | 0.4 | 0.733 | 0.25 | 0.373 | 0.8 |
| **SVM** | DECT_habitat_c-train | 0.962 | 0.973 | 0.9345 - 1.0000 | 0.985 | 0.919 | 0.957 | 0.971 | 0.957 | 0.985 | 0.971 | 0.571 |
|  | DECT_habitat_c-test | 0.848 | 0.953 | 0.8990 - 1.0000 | 0.8 | 1 | 1 | 0.611 | 1 | 0.8 | 0.889 | 0.49 |
|  | DECT_habitat_c-val | 0.814 | 0.848 | 0.7524 - 0.9434 | 0.909 | 0.654 | 0.816 | 0.81 | 0.816 | 0.909 | 0.86 | 0.355 |
|  | DECT_habitat_d-train | 0.943 | 0.993 | 0.9837 - 1.0000 | 0.941 | 0.946 | 0.97 | 0.897 | 0.97 | 0.941 | 0.955 | 0.645 |
|  | DECT_habitat_d-test | 0.848 | 0.719 | 0.5267 - 0.9123 | 0.971 | 0.455 | 0.85 | 0.833 | 0.85 | 0.971 | 0.907 | 0.417 |
|  | DECT_habitat_d-val | 0.686 | 0.648 | 0.5067 - 0.7897 | 0.773 | 0.538 | 0.739 | 0.583 | 0.739 | 0.773 | 0.756 | 0.518 |
|  | DECT_habitats-train | 0.943 | 0.993 | 0.9844 - 1.0000 | 0.912 | 1 | 1 | 0.86 | 1 | 0.912 | 0.954 | 0.843 |
|  | DECT_habitats-test | 0.826 | 0.888 | 0.7841 - 0.9925 | 0.8 | 0.909 | 0.966 | 0.588 | 0.966 | 0.8 | 0.875 | 0.5 |
|  | DECT_habitats-val | 0.757 | 0.745 | 0.6223 - 0.8672 | 0.773 | 0.731 | 0.829 | 0.655 | 0.829 | 0.773 | 0.8 | 0.551 |
|  | DECT_radiomics-train | 0.971 | 0.975 | 0.9315 - 1.0000 | 0.985 | 0.946 | 0.971 | 0.972 | 0.971 | 0.985 | 0.978 | 0.583 |
|  | DECT_radiomics-test | 0.761 | 0.818 | 0.6723 - 0.9640 | 0.714 | 0.909 | 0.962 | 0.5 | 0.962 | 0.714 | 0.82 | 0.635 |
|  | DECT_radiomics-val | 0.7 | 0.68 | 0.5366 - 0.8235 | 0.682 | 0.731 | 0.811 | 0.576 | 0.811 | 0.682 | 0.741 | 0.658 |
|  | CT_radiomics-val | 0.676 | 0.764 | 0.6402 - 0.8885 | 0.653 | 0.727 | 0.842 | 0.485 | 0.842 | 0.653 | 0.736 | 0.5 |
|  | CT_habitat_a-test | 0.607 | 0.636 | 0.4882 - 0.7840 | 0.387 | 0.88 | 0.8 | 0.537 | 0.8 | 0.387 | 0.522 | 0.824 |
|  | CT_habitat_a-train | 0.898 | 0.955 | 0.9169 - 0.9930 | 0.9 | 0.896 | 0.935 | 0.843 | 0.935 | 0.9 | 0.917 | 0.67 |
|  | CT_habitat_a-val | 0.704 | 0.681 | 0.5428 - 0.8190 | 0.714 | 0.682 | 0.833 | 0.517 | 0.833 | 0.714 | 0.769 | 0.626 |
|  | CT_habitat_b-test | 0.804 | 0.868 | 0.7764 - 0.9604 | 0.742 | 0.88 | 0.885 | 0.733 | 0.885 | 0.742 | 0.807 | 0.578 |
|  | CT_habitat_b-train | 0.875 | 0.918 | 0.8635 - 0.9724 | 0.837 | 0.937 | 0.957 | 0.776 | 0.957 | 0.837 | 0.893 | 0.719 |
|  | CT_habitat_b-val | 0.789 | 0.817 | 0.7036 - 0.9309 | 0.755 | 0.864 | 0.925 | 0.613 | 0.925 | 0.755 | 0.831 | 0.628 |
|  | CT_habitats-test | 0.804 | 0.837 | 0.7308 - 0.9440 | 0.742 | 0.88 | 0.885 | 0.733 | 0.885 | 0.742 | 0.807 | 0.713 |
|  | CT_habitats-train | 0.906 | 0.96 | 0.9284 - 0.9924 | 0.937 | 0.854 | 0.915 | 0.891 | 0.915 | 0.937 | 0.926 | 0.609 |
|  | CT_habitats-val | 0.803 | 0.803 | 0.6845 - 0.9222 | 0.898 | 0.591 | 0.83 | 0.722 | 0.83 | 0.898 | 0.863 | 0.405 |
|  | CT_radiomics-train | 0.938 | 0.971 | 0.9374 - 1.0000 | 0.962 | 0.896 | 0.939 | 0.935 | 0.939 | 0.962 | 0.951 | 0.579 |
|  | CT_radiomics-test | 0.821 | 0.843 | 0.7315 - 0.9537 | 0.71 | 0.96 | 0.957 | 0.727 | 0.957 | 0.71 | 0.815 | 0.674 |
|  | CT_radiomics-val | 0.662 | 0.792 | 0.6875 - 0.8969 | 0.551 | 0.909 | 0.931 | 0.476 | 0.931 | 0.551 | 0.692 | 0.784 |
| **XGBoost** | CT_habitat_a-test | 0.643 | 0.721 | 0.5873 - 0.8540 | 0.484 | 0.84 | 0.789 | 0.568 | 0.789 | 0.484 | 0.6 | 0.794 |
|  | CT_habitat_a-train | 0.984 | 1 | 0.9990 - 1.0000 | 0.975 | 1 | 1 | 0.96 | 1 | 0.975 | 0.987 | 0.551 |
|  | CT_habitat_a-val | 0.563 | 0.587 | 0.4420 - 0.7315 | 0.469 | 0.773 | 0.821 | 0.395 | 0.821 | 0.469 | 0.597 | 0.818 |
|  | CT_habitat_b-test | 0.804 | 0.848 | 0.7389 - 0.9566 | 0.742 | 0.88 | 0.885 | 0.733 | 0.885 | 0.742 | 0.807 | 0.708 |
|  | CT_habitat_b-train | 0.984 | 0.999 | 0.9959 - 1.0000 | 0.987 | 0.979 | 0.987 | 0.979 | 0.987 | 0.987 | 0.987 | 0.523 |
|  | CT_habitat_b-val | 0.732 | 0.805 | 0.6874 - 0.9230 | 0.694 | 0.818 | 0.895 | 0.545 | 0.895 | 0.694 | 0.782 | 0.614 |
|  | CT_habitats-test | 0.75 | 0.762 | 0.6306 - 0.8933 | 0.742 | 0.76 | 0.793 | 0.704 | 0.793 | 0.742 | 0.767 | 0.676 |
|  | CT_habitats-train | 0.977 | 0.999 | 0.9976 - 1.0000 | 0.962 | 1 | 1 | 0.941 | 1 | 0.962 | 0.981 | 0.568 |
|  | CT_habitats-val | 0.676 | 0.677 | 0.5345 - 0.8199 | 0.673 | 0.682 | 0.825 | 0.484 | 0.825 | 0.673 | 0.742 | 0.626 |
|  | CT_radiomics-train | 0.984 | 0.999 | 0.9982 - 1.0000 | 0.975 | 1 | 1 | 0.96 | 1 | 0.975 | 0.987 | 0.671 |
|  | CT_radiomics-test | 0.714 | 0.72 | 0.5823 - 0.8577 | 0.645 | 0.8 | 0.8 | 0.645 | 0.8 | 0.645 | 0.714 | 0.749 |
|  | CT_radiomics-val | 0.704 | 0.759 | 0.6371 - 0.8805 | 0.653 | 0.818 | 0.889 | 0.514 | 0.889 | 0.653 | 0.753 | 0.756 |
|  | DECT_habitat_c-train | 0.99 | 1 | 1.0000 - 1.0000 | 0.985 | 1 | 1 | 0.974 | 1 | 0.985 | 0.993 | 0.754 |
|  | DECT_habitat_c-test | 0.783 | 0.849 | 0.7355 - 0.9632 | 0.771 | 0.818 | 0.931 | 0.529 | 0.931 | 0.771 | 0.844 | 0.568 |
|  | DECT_habitat_c-val | 0.714 | 0.723 | 0.5955 - 0.8503 | 0.659 | 0.808 | 0.853 | 0.583 | 0.853 | 0.659 | 0.744 | 0.658 |
|  | DECT_habitat_d-train | 0.99 | 1 | 1.0000 - 1.0000 | 0.985 | 1 | 1 | 0.974 | 1 | 0.985 | 0.993 | 0.759 |
|  | DECT_habitat_d-test | 0.761 | 0.553 | 0.3356 - 0.7709 | 0.914 | 0.273 | 0.8 | 0.5 | 0.8 | 0.914 | 0.853 | 0.558 |
|  | DECT_habitat_d-val | 0.657 | 0.608 | 0.4650 - 0.7518 | 0.75 | 0.5 | 0.717 | 0.542 | 0.717 | 0.75 | 0.733 | 0.698 |
|  | DECT_habitats-train | 0.99 | 1 | 1.0000 - 1.0000 | 0.985 | 1 | 1 | 0.974 | 1 | 0.985 | 0.993 | 0.741 |
|  | DECT_habitats-test | 0.696 | 0.795 | 0.6586 - 0.9310 | 0.657 | 0.818 | 0.92 | 0.429 | 0.92 | 0.657 | 0.767 | 0.772 |
|  | DECT_habitats-val | 0.643 | 0.664 | 0.5342 - 0.7936 | 0.523 | 0.846 | 0.852 | 0.512 | 0.852 | 0.523 | 0.648 | 0.81 |
|  | DECT_radiomics-train | 0.99 | 1 | 1.0000 - 1.0000 | 0.985 | 1 | 1 | 0.974 | 1 | 0.985 | 0.993 | 0.722 |
|  | DECT_radiomics-test | 0.87 | 0.857 | 0.7011 - 1.0000 | 0.886 | 0.818 | 0.939 | 0.692 | 0.939 | 0.886 | 0.912 | 0.552 |
|  | DECT_radiomics-val | 0.586 | 0.567 | 0.4310 - 0.7027 | 0.545 | 0.654 | 0.727 | 0.459 | 0.727 | 0.545 | 0.623 | 0.829 |

**Table S4:** The performance of different classifiers in predicting clinical outcomes

| **Classifiers** | **Cohorts** | **Accuracy** | **AUC** | **95% CI** | **Sensitivity** | **Specificity** | **PPV** | **NPV** | **Precision** | **Recall** | **F1** | **Threshold** |
| --- | --- | --- | --- | --- | --- | --- | --- | --- | --- | --- | --- | --- |
| **ExtraTrees** | CT_habitat_a-train | 0.617 | 1 | 1.0000 - 1.0000 | 0 | 1 | 0 | 0.617 | 0 | 0 | NaN | 1 |
|  | CT_habitat_a-test | 0.554 | 0.672 | 0.5233 - 0.8203 | 0.455 | 0.618 | 0.435 | 0.636 | 0.435 | 0.455 | 0.444 | 0.3 |
|  | CT_habitat_a-val | 0.662 | 0.664 | 0.5354 - 0.7931 | 0.4 | 0.854 | 0.667 | 0.66 | 0.667 | 0.4 | 0.5 | 0.4 |
|  | CT_habitat_b-train | 0.617 | 1 | 1.0000 - 1.0000 | 0 | 1 | 0 | 0.617 | 0 | 0 | NaN | 1 |
|  | CT_habitat_b-test | 0.625 | 0.666 | 0.5238 - 0.8077 | 0.727 | 0.559 | 0.516 | 0.76 | 0.516 | 0.727 | 0.604 | 0.3 |
|  | CT_habitat_b-val | 0.704 | 0.7 | 0.5671 - 0.8329 | 0.3 | 1 | 1 | 0.661 | 1 | 0.3 | 0.462 | 0.7 |
|  | CT_habitats-train | 0.617 | 1 | 1.0000 - 1.0000 | 0 | 1 | 0 | 0.617 | 0 | 0 | NaN | 1 |
|  | CT_habitats-test | 0.607 | 0.692 | 0.5529 - 0.8308 | 0.545 | 0.647 | 0.5 | 0.687 | 0.5 | 0.545 | 0.522 | 0.4 |
|  | CT_habitats-val | 0.718 | 0.727 | 0.6014 - 0.8523 | 0.367 | 0.976 | 0.917 | 0.678 | 0.917 | 0.367 | 0.524 | 0.6 |
|  | CT_radiomics-train | 0.617 | 1 | 1.0000 - 1.0000 | 0 | 1 | 0 | 0.617 | 0 | 0 | NaN | 1 |
|  | CT_radiomics-test | 0.679 | 0.734 | 0.6028 - 0.8651 | 0.682 | 0.676 | 0.577 | 0.767 | 0.577 | 0.682 | 0.625 | 0.4 |
|  | CT_radiomics-val | 0.69 | 0.687 | 0.5607 - 0.8133 | 0.467 | 0.854 | 0.7 | 0.686 | 0.7 | 0.467 | 0.56 | 0.4 |
|  | DECT_habitat_c-train | 0.648 | 1 | 1.0000 - 1.0000 | 0 | 1 | 0 | 0.648 | 0 | 0 | NaN | 1 |
|  | DECT_habitat_c-test | 0.783 | 0.83 | 0.7056 - 0.9548 | 0.375 | 1 | 1 | 0.75 | 1 | 0.375 | 0.545 | 0.6 |
|  | DECT_habitat_c-val | 0.8 | 0.846 | 0.7433 - 0.9496 | 0.714 | 0.837 | 0.652 | 0.872 | 0.652 | 0.714 | 0.682 | 0.5 |
|  | DECT_habitat_d-train | 0.648 | 1 | 1.0000 - 1.0000 | 0 | 1 | 0 | 0.648 | 0 | 0 | NaN | 1 |
|  | DECT_habitat_d-test | 0.478 | 0.497 | 0.3192 - 0.6745 | 0.562 | 0.433 | 0.346 | 0.65 | 0.346 | 0.562 | 0.429 | 0.3 |
|  | DECT_habitat_d-val | 0.586 | 0.601 | 0.4597 - 0.7424 | 0.524 | 0.612 | 0.367 | 0.75 | 0.367 | 0.524 | 0.431 | 0.3 |
|  | DECT_habitats-train | 0.648 | 1 | 1.0000 - 1.0000 | 0 | 1 | 0 | 0.648 | 0 | 0 | NaN | 1.00E+00 |
|  | DECT_habitats-test | 0.717 | 0.782 | 0.6439 - 0.9206 | 0.437 | 0.867 | 0.636 | 0.743 | 0.636 | 0.437 | 0.519 | 5.00E-01 |
|  | DECT_habitats-val | 0.671 | 0.663 | 0.5231 - 0.8034 | 0.238 | 0.857 | 0.417 | 0.724 | 0.417 | 0.238 | 0.303 | 5.00E-01 |
|  | DECT_radiomics-train | 0.648 | 1 | 1.0000 - 1.0000 | 0 | 1 | 0 | 0.648 | 0 | 0 | NaN | 1 |
|  | DECT_radiomics-test | 0.696 | 0.676 | 0.5127 - 0.8394 | 0.187 | 0.967 | 0.75 | 0.69 | 0.75 | 0.187 | 0.3 | 0.6 |
|  | DECT_radiomics-val | 0.7 | 0.703 | 0.5643 - 0.8419 | 0.524 | 0.776 | 0.5 | 0.792 | 0.5 | 0.524 | 5.12E-01 | 0.4 |
| **KNN** | CT_habitat_a-train | 0.859 | 0.926 | 0.8852 - 0.9668 | 0.735 | 0.937 | 0.878 | 0.851 | 0.878 | 0.735 | 0.8 | 0.4 |
|  | CT_habitat_a-test | 0.625 | 0.616 | 0.4669 - 0.7657 | 0.273 | 0.853 | 0.545 | 0.644 | 0.545 | 0.273 | 0.364 | 0.4 |
|  | CT_habitat_a-val | 0.62 | 0.57 | 0.4386 - 0.7013 | 0.1 | 1 | 1 | 0.603 | 1 | 0.1 | 0.182 | 0.6 |
|  | CT_habitat_b-train | 0.836 | 0.922 | 0.8797 - 0.9640 | 0.653 | 0.949 | 0.889 | 0.815 | 0.889 | 0.653 | 0.753 | 0.4 |
|  | CT_habitat_b-test | 0.607 | 0.632 | 0.4852 - 0.7781 | 0.455 | 0.706 | 0.5 | 0.667 | 0.5 | 0.455 | 0.476 | 0.4 |
|  | CT_habitat_b-val | 0.69 | 0.667 | 0.5345 - 0.7989 | 0.333 | 0.951 | 0.833 | 0.661 | 0.833 | 0.333 | 0.476 | 0.6 |
|  | CT_habitats-train | 0.812 | 0.931 | 0.8911 - 0.9715 | 0.51 | 1 | 1 | 0.767 | 1 | 0.51 | 0.676 | 0.6 |
|  | CT_habitats-test | 0.643 | 0.658 | 0.5145 - 0.8024 | 0.318 | 0.853 | 0.583 | 0.659 | 0.583 | 0.318 | 0.412 | 0.4 |
|  | CT_habitats-val | 0.606 | 0.65 | 0.5175 - 0.7817 | 0.067 | 1 | 1 | 0.594 | 1 | 0.067 | 0.125 | 0.8 |
|  | CT_radiomics-train | 0.836 | 0.922 | 0.8802 - 0.9643 | 0.673 | 0.937 | 0.868 | 0.822 | 0.868 | 0.673 | 0.759 | 0.4 |
|  | CT_radiomics-test | 0.661 | 0.731 | 0.5954 - 0.8658 | 0.409 | 0.824 | 0.6 | 0.683 | 0.6 | 0.409 | 0.486 | 0.4 |
|  | CT_radiomics-val | 0.662 | 0.693 | 0.5736 - 0.8126 | 0.5 | 0.78 | 0.625 | 0.681 | 0.625 | 0.5 | 0.556 | 0.2 |
|  | DECT_habitat_c-train | 0.838 | 0.931 | 0.8878 - 0.9735 | 0.568 | 0.985 | 0.955 | 0.807 | 0.955 | 0.568 | 0.712 | 0.4 |
|  | DECT_habitat_c-test | 0.674 | 0.74 | 0.5953 - 0.8838 | 0.687 | 0.667 | 0.524 | 0.8 | 0.524 | 0.687 | 0.595 | 0.2 |
|  | DECT_habitat_c-val | 0.7 | 0.744 | 0.6305 - 0.8583 | 0.476 | 0.796 | 0.5 | 0.78 | 0.5 | 0.476 | 0.488 | 0.4 |
|  | DECT_habitat_d-train | 0.781 | 0.862 | 0.7960 - 0.9290 | 0.541 | 0.912 | 0.769 | 0.785 | 0.769 | 0.541 | 0.635 | 0.4 |
|  | DECT_habitat_d-test | 0.63 | 0.415 | 0.2367 - 0.5925 | 0 | 0.967 | 0 | 0.644 | 0 | 0 | NaN | 0.6 |
|  | DECT_habitat_d-val | 0.671 | 0.466 | 0.3157 - 0.6173 | 0 | 0.959 | 0 | 0.691 | 0 | 0 | NaN | 0.6 |
|  | DECT_habitats-train | 0.829 | 0.915 | 0.8646 - 0.9653 | 0.622 | 0.941 | 0.852 | 0.821 | 0.852 | 0.622 | 0.719 | 4.00E-01 |
|  | DECT_habitats-test | 0.696 | 0.758 | 0.6141 - 0.9026 | 0.312 | 0.9 | 0.625 | 0.711 | 0.625 | 0.312 | 0.417 | 6.00E-01 |
|  | DECT_habitats-val | 0.743 | 0.812 | 0.6909 - 0.9340 | 0.286 | 0.939 | 0.667 | 0.754 | 0.667 | 0.286 | 0.4 | 6.00E-01 |
|  | DECT_radiomics-train | 0.819 | 0.859 | 0.7883 - 0.9295 | 0.622 | 0.926 | 0.821 | 0.818 | 0.821 | 0.622 | 0.708 | 0.4 |
|  | DECT_radiomics-test | 0.761 | 0.78 | 0.6390 - 0.9214 | 0.562 | 0.867 | 0.692 | 0.788 | 0.692 | 0.562 | 0.621 | 0.4 |
|  | DECT_radiomics-val | 0.714 | 0.646 | 0.4972 - 0.7953 | 0.286 | 0.898 | 0.545 | 0.746 | 0.545 | 0.286 | 3.75E-01 | 0.6 |
| **LightGBM** | CT_habitat_a-train | 0.906 | 0.958 | 0.9279 - 0.9874 | 0.816 | 0.962 | 0.93 | 0.894 | 0.93 | 0.816 | 0.87 | 0.432 |
|  | CT_habitat_a-test | 0.643 | 0.686 | 0.5422 - 0.8308 | 0.591 | 0.676 | 0.542 | 0.719 | 0.542 | 0.591 | 0.565 | 0.339 |
|  | CT_habitat_a-val | 0.549 | 0.602 | 0.4688 - 0.7361 | 0.733 | 0.415 | 0.478 | 0.68 | 0.478 | 0.733 | 0.579 | 0.293 |
|  | CT_habitat_b-train | 0.805 | 0.903 | 0.8520 - 0.9540 | 0.796 | 0.81 | 0.722 | 0.865 | 0.722 | 0.796 | 0.757 | 0.413 |
|  | CT_habitat_b-test | 0.625 | 0.65 | 0.4991 - 0.8018 | 0.727 | 0.559 | 0.516 | 0.76 | 0.516 | 0.727 | 0.604 | 0.269 |
|  | CT_habitat_b-val | 0.577 | 0.638 | 0.5091 - 0.7673 | 0.767 | 0.439 | 0.5 | 0.72 | 0.5 | 0.767 | 0.605 | 0.28 |
|  | CT_habitats-train | 0.891 | 0.966 | 0.9400 - 0.9926 | 0.878 | 0.899 | 0.843 | 0.922 | 0.843 | 0.878 | 0.86 | 0.396 |
|  | CT_habitats-test | 0.696 | 0.636 | 0.4804 - 0.7910 | 0.273 | 0.971 | 0.857 | 0.673 | 0.857 | 0.273 | 0.414 | 0.615 |
|  | CT_habitats-val | 0.69 | 0.675 | 0.5441 - 0.8055 | 0.4 | 0.902 | 0.75 | 0.673 | 0.75 | 0.4 | 0.522 | 0.531 |
|  | CT_radiomics-train | 0.852 | 0.954 | 0.9237 - 0.9844 | 0.918 | 0.81 | 0.75 | 0.941 | 0.75 | 0.918 | 0.826 | 0.389 |
|  | CT_radiomics-test | 0.679 | 0.717 | 0.5838 - 0.8507 | 0.727 | 0.647 | 0.571 | 0.786 | 0.571 | 0.727 | 0.64 | 0.347 |
|  | CT_radiomics-val | 0.634 | 0.657 | 0.5261 - 0.7877 | 0.733 | 0.561 | 0.55 | 0.742 | 0.55 | 0.733 | 0.629 | 0.275 |
|  | DECT_habitat_c-train | 0.876 | 0.947 | 0.9068 - 0.9874 | 0.919 | 0.853 | 0.773 | 0.951 | 0.773 | 0.919 | 0.84 | 0.378 |
|  | DECT_habitat_c-test | 0.609 | 0.639 | 0.4758 - 0.8013 | 0.812 | 0.5 | 0.464 | 0.833 | 0.464 | 0.812 | 0.591 | 0.259 |
|  | DECT_habitat_c-val | 0.457 | 0.574 | 0.4356 - 0.7131 | 0.952 | 0.245 | 0.351 | 0.923 | 0.351 | 0.952 | 0.513 | 0.197 |
|  | DECT_habitat_d-train | 0.819 | 0.865 | 0.7813 - 0.9488 | 0.676 | 0.897 | 0.781 | 0.836 | 0.781 | 0.676 | 0.725 | 0.387 |
|  | DECT_habitat_d-test | 0.587 | 0.357 | 0.1738 - 0.5408 | 0.187 | 0.8 | 0.333 | 0.649 | 0.333 | 0.187 | 0.24 | 0.41 |
|  | DECT_habitat_d-val | 0.514 | 0.523 | 0.3808 - 0.6658 | 0.714 | 0.429 | 0.349 | 0.778 | 0.349 | 0.714 | 0.469 | 0.255 |
|  | DECT_habitats-train | 0.905 | 0.964 | 0.9322 - 0.9963 | 0.865 | 0.926 | 0.865 | 0.926 | 0.865 | 0.865 | 0.865 | 3.82E-01 |
|  | DECT_habitats-test | 0.761 | 0.769 | 0.6249 - 0.9126 | 0.625 | 0.833 | 0.667 | 0.806 | 0.667 | 0.625 | 0.645 | 3.39E-01 |
|  | DECT_habitats-val | 0.557 | 0.636 | 0.4934 - 0.7777 | 0.762 | 0.469 | 0.381 | 0.821 | 0.381 | 0.762 | 0.508 | 3.73E-01 |
|  | DECT_radiomics-train | 0.895 | 0.947 | 0.9064 - 0.9878 | 0.919 | 0.882 | 0.81 | 0.952 | 0.81 | 0.919 | 0.861 | 0.359 |
|  | DECT_radiomics-test | 0.717 | 0.616 | 0.4340 - 0.7973 | 0.312 | 0.933 | 0.714 | 0.718 | 0.714 | 0.312 | 0.435 | 0.401 |
|  | DECT_radiomics-val | 0.657 | 0.59 | 0.4399 - 0.7409 | 0.571 | 0.694 | 0.444 | 0.791 | 0.444 | 0.571 | 5.00E-01 | 0.334 |
| **LR** | CT_habitat_a-train | 0.898 | 0.919 | 0.8604 - 0.9769 | 0.816 | 0.949 | 0.909 | 0.893 | 0.909 | 0.816 | 0.86 | 0.497 |
|  | CT_habitat_a-test | 0.768 | 0.845 | 0.7409 - 0.9490 | 0.818 | 0.735 | 0.667 | 0.862 | 0.667 | 0.818 | 0.735 | 0.317 |
|  | CT_habitat_a-val | 0.775 | 0.82 | 0.7185 - 0.9222 | 0.633 | 0.878 | 0.792 | 0.766 | 0.792 | 0.633 | 0.704 | 0.563 |
|  | CT_habitat_b-train | 0.875 | 0.914 | 0.8620 - 0.9670 | 0.694 | 0.987 | 0.971 | 0.839 | 0.971 | 0.694 | 0.81 | 0.648 |
|  | CT_habitat_b-test | 0.714 | 0.702 | 0.5612 - 0.8426 | 0.364 | 0.941 | 0.8 | 0.696 | 0.8 | 0.364 | 0.5 | 0.829 |
|  | CT_habitat_b-val | 0.662 | 0.597 | 0.4577 - 0.7358 | 0.4 | 0.854 | 0.667 | 0.66 | 0.667 | 0.4 | 0.5 | 0.693 |
|  | CT_habitats-train | 0.93 | 0.964 | 0.9334 - 0.9943 | 0.837 | 0.987 | 0.976 | 0.907 | 0.976 | 0.837 | 0.901 | 0.503 |
|  | CT_habitats-test | 0.768 | 0.809 | 0.6867 - 0.9309 | 0.727 | 0.794 | 0.696 | 0.818 | 0.696 | 0.727 | 0.711 | 0.445 |
|  | CT_habitats-val | 0.704 | 0.731 | 0.6078 - 0.8540 | 0.633 | 0.756 | 0.655 | 0.738 | 0.655 | 0.633 | 0.644 | 0.374 |
|  | CT_radiomics-train | 0.938 | 0.968 | 0.9365 - 0.9995 | 0.918 | 0.949 | 0.918 | 0.949 | 0.918 | 0.918 | 0.918 | 0.401 |
|  | CT_radiomics-test | 0.75 | 0.817 | 0.7061 - 0.9275 | 0.818 | 0.706 | 0.643 | 0.857 | 0.643 | 0.818 | 0.72 | 0.531 |
|  | CT_radiomics-val | 0.704 | 0.759 | 0.6483 - 0.8704 | 0.7 | 0.707 | 0.636 | 0.763 | 0.636 | 0.7 | 0.667 | 0.348 |
|  | DECT_habitat_c-train | 0.905 | 0.946 | 0.9047 - 0.9872 | 0.73 | 1 | 1 | 0.872 | 1 | 0.73 | 0.844 | 0.592 |
|  | DECT_habitat_c-test | 0.804 | 0.854 | 0.7391 - 0.9692 | 0.687 | 0.867 | 0.733 | 0.839 | 0.733 | 0.687 | 0.71 | 0.571 |
|  | DECT_habitat_c-val | 0.843 | 0.827 | 0.7091 - 0.9449 | 0.571 | 0.959 | 0.857 | 0.839 | 0.857 | 0.571 | 0.686 | 0.67 |
|  | DECT_habitat_d-train | 0.781 | 0.751 | 0.6502 - 0.8522 | 0.486 | 0.941 | 0.818 | 0.771 | 0.818 | 0.486 | 0.61 | 0.462 |
|  | DECT_habitat_d-test | 0.543 | 0.417 | 0.2341 - 0.5992 | 0.312 | 0.667 | 0.333 | 0.645 | 0.333 | 0.312 | 0.323 | 0.417 |
|  | DECT_habitat_d-val | 0.514 | 0.65 | 0.5133 - 0.7870 | 0.857 | 0.367 | 0.367 | 0.857 | 0.367 | 0.857 | 0.514 | 0.256 |
|  | DECT_habitats-train | 0.886 | 0.953 | 0.9149 - 0.9906 | 0.865 | 0.897 | 0.821 | 0.924 | 0.821 | 0.865 | 0.842 | 3.81E-01 |
|  | DECT_habitats-test | 0.717 | 0.779 | 0.6424 - 0.9159 | 0.687 | 0.733 | 0.579 | 0.815 | 0.579 | 0.687 | 0.629 | 3.68E-01 |
|  | DECT_habitats-val | 0.814 | 0.869 | 0.7623 - 0.9753 | 0.81 | 0.816 | 0.654 | 0.909 | 0.654 | 0.81 | 0.723 | 4.12E-01 |
|  | DECT_radiomics-train | 0.895 | 0.936 | 0.8868 - 0.9844 | 0.784 | 0.956 | 0.906 | 0.89 | 0.906 | 0.784 | 0.841 | 0.523 |
|  | DECT_radiomics-test | 0.761 | 0.838 | 0.7201 - 0.9549 | 0.75 | 0.767 | 0.632 | 0.852 | 0.632 | 0.75 | 0.686 | 0.304 |
|  | DECT_radiomics-val | 0.8 | 0.781 | 0.6427 - 0.9200 | 0.571 | 0.898 | 0.706 | 0.83 | 0.706 | 0.571 | 6.32E-01 | 0.688 |
| **MLP** | CT_habitat_a-train | 0.852 | 0.906 | 0.8484 - 0.9630 | 0.878 | 0.835 | 0.768 | 0.917 | 0.768 | 0.878 | 0.819 | 0.36 |
|  | CT_habitat_a-test | 0.661 | 0.726 | 0.5922 - 0.8597 | 0.773 | 0.588 | 0.548 | 0.8 | 0.548 | 0.773 | 0.642 | 0.324 |
|  | CT_habitat_a-val | 0.704 | 0.682 | 0.5506 - 0.8137 | 0.667 | 0.732 | 0.645 | 0.75 | 0.645 | 0.667 | 0.656 | 0.361 |
|  | CT_habitat_b-train | 0.852 | 0.893 | 0.8315 - 0.9552 | 0.735 | 0.924 | 0.857 | 0.849 | 0.857 | 0.735 | 0.791 | 0.429 |
|  | CT_habitat_b-test | 0.714 | 0.684 | 0.5359 - 0.8331 | 0.545 | 0.824 | 0.667 | 0.737 | 0.667 | 0.545 | 0.6 | 0.434 |
|  | CT_habitat_b-val | 0.662 | 0.654 | 0.5221 - 0.7852 | 0.4 | 0.854 | 0.667 | 0.66 | 0.667 | 0.4 | 0.5 | 0.449 |
|  | CT_habitats-train | 0.914 | 0.957 | 0.9163 - 0.9984 | 0.857 | 0.949 | 0.913 | 0.915 | 0.913 | 0.857 | 0.884 | 0.409 |
|  | CT_habitats-test | 0.732 | 0.761 | 0.6308 - 0.8906 | 0.591 | 0.824 | 0.684 | 0.757 | 0.684 | 0.591 | 0.634 | 0.469 |
|  | CT_habitats-val | 0.648 | 0.675 | 0.5469 - 0.8027 | 0.633 | 0.659 | 0.576 | 0.711 | 0.576 | 0.633 | 0.603 | 0.367 |
|  | CT_radiomics-train | 0.867 | 0.94 | 0.8992 - 0.9814 | 0.837 | 0.886 | 0.82 | 0.897 | 0.82 | 0.837 | 0.828 | 0.393 |
|  | CT_radiomics-test | 0.768 | 0.803 | 0.6832 - 0.9237 | 0.773 | 0.765 | 0.68 | 0.839 | 0.68 | 0.773 | 0.723 | 0.412 |
|  | CT_radiomics-val | 0.761 | 0.781 | 0.6705 - 0.8921 | 0.633 | 0.854 | 0.76 | 0.761 | 0.76 | 0.633 | 0.691 | 0.407 |
|  | DECT_habitat_c-train | 0.829 | 0.931 | 0.8856 - 0.9769 | 0.892 | 0.794 | 0.702 | 0.931 | 0.702 | 0.892 | 0.786 | 0.358 |
|  | DECT_habitat_c-test | 0.739 | 0.813 | 0.6716 - 0.9534 | 0.812 | 0.7 | 0.591 | 0.875 | 0.591 | 0.812 | 0.684 | 0.325 |
|  | DECT_habitat_c-val | 0.714 | 0.765 | 0.6403 - 0.8893 | 0.714 | 0.714 | 0.517 | 0.854 | 0.517 | 0.714 | 0.6 | 0.364 |
|  | DECT_habitat_d-train | 0.752 | 0.744 | 0.6409 - 0.8479 | 0.622 | 0.824 | 0.657 | 0.8 | 0.657 | 0.622 | 0.639 | 0.348 |
|  | DECT_habitat_d-test | 0.652 | 0.454 | 0.2620 - 0.6463 | 0.062 | 0.967 | 0.5 | 0.659 | 0.5 | 0.062 | 0.111 | 0.536 |
|  | DECT_habitat_d-val | 0.543 | 0.671 | 0.5294 - 0.8117 | 0.905 | 0.388 | 0.388 | 0.905 | 0.388 | 0.905 | 0.543 | 0.319 |
|  | DECT_habitats-train | 0.867 | 0.948 | 0.9108 - 0.9858 | 0.946 | 0.824 | 0.745 | 0.966 | 0.745 | 0.946 | 0.833 | 3.46E-01 |
|  | DECT_habitats-test | 0.804 | 0.787 | 0.6369 - 0.9381 | 0.562 | 0.933 | 0.818 | 0.8 | 0.818 | 0.562 | 0.667 | 5.23E-01 |
|  | DECT_habitats-val | 0.814 | 0.864 | 0.7580 - 0.9699 | 0.714 | 0.857 | 0.682 | 0.875 | 0.682 | 0.714 | 0.698 | 3.99E-01 |
|  | DECT_radiomics-train | 0.895 | 0.94 | 0.8941 - 0.9850 | 0.811 | 0.941 | 0.882 | 0.901 | 0.882 | 0.811 | 0.845 | 0.43 |
|  | DECT_radiomics-test | 0.696 | 0.773 | 0.6367 - 0.9091 | 0.75 | 0.667 | 0.545 | 0.833 | 0.545 | 0.75 | 0.632 | 0.329 |
|  | DECT_radiomics-val | 0.8 | 0.776 | 0.6346 - 0.9184 | 0.571 | 0.898 | 0.706 | 0.83 | 0.706 | 0.571 | 6.32E-01 | 0.565 |
| **RandomForest** | CT_habitat_a-train | 0.969 | 0.997 | 0.9936 - 1.0000 | 0.939 | 0.987 | 0.979 | 0.963 | 0.979 | 0.939 | 0.958 | 0.5 |
|  | CT_habitat_a-test | 0.661 | 0.628 | 0.4726 - 0.7828 | 0.318 | 0.882 | 0.636 | 0.667 | 0.636 | 0.318 | 0.424 | 0.5 |
|  | CT_habitat_a-val | 0.662 | 0.691 | 0.5676 - 0.8137 | 0.3 | 0.927 | 0.75 | 0.644 | 0.75 | 0.3 | 0.429 | 0.5 |
|  | CT_habitat_b-train | 0.969 | 0.998 | 0.9940 - 1.0000 | 0.959 | 0.975 | 0.959 | 0.975 | 0.959 | 0.959 | 0.959 | 0.4 |
|  | CT_habitat_b-test | 0.643 | 0.601 | 0.4432 - 0.7587 | 0.273 | 0.882 | 0.6 | 0.652 | 0.6 | 0.273 | 0.375 | 0.6 |
|  | CT_habitat_b-val | 0.676 | 0.715 | 0.5928 - 0.8373 | 0.4 | 0.878 | 0.706 | 0.667 | 0.706 | 0.4 | 0.511 | 0.6 |
|  | CT_habitats-train | 0.984 | 1 | 0.9987 - 1.0000 | 0.959 | 1 | 1 | 0.975 | 1 | 0.959 | 0.979 | 0.5 |
|  | CT_habitats-test | 0.661 | 0.675 | 0.5284 - 0.8219 | 0.409 | 0.824 | 0.6 | 0.683 | 0.6 | 0.409 | 0.486 | 0.5 |
|  | CT_habitats-val | 0.62 | 0.675 | 0.5493 - 0.8003 | 0.467 | 0.732 | 0.56 | 0.652 | 0.56 | 0.467 | 0.509 | 0.6 |
|  | CT_radiomics-train | 0.961 | 0.998 | 0.9939 - 1.0000 | 0.939 | 0.975 | 0.958 | 0.962 | 0.958 | 0.939 | 0.948 | 0.5 |
|  | CT_radiomics-test | 0.554 | 0.6 | 0.4539 - 0.7466 | 0.727 | 0.441 | 0.457 | 0.714 | 0.457 | 0.727 | 0.561 | 0.3 |
|  | CT_radiomics-val | 0.676 | 0.736 | 0.6187 - 0.8537 | 0.333 | 0.927 | 0.769 | 0.655 | 0.769 | 0.333 | 0.465 | 0.5 |
|  | DECT_habitat_c-train | 0.962 | 0.999 | 0.9975 - 1.0000 | 0.892 | 1 | 1 | 0.944 | 1 | 0.892 | 0.943 | 0.6 |
|  | DECT_habitat_c-test | 0.609 | 0.726 | 0.5752 - 0.8769 | 0.375 | 0.733 | 0.429 | 0.687 | 0.429 | 0.375 | 0.4 | 0.4 |
|  | DECT_habitat_c-val | 0.571 | 0.677 | 0.5524 - 0.8023 | 0.667 | 0.531 | 0.378 | 0.788 | 0.378 | 0.667 | 0.483 | 0.3 |
|  | DECT_habitat_d-train | 0.962 | 0.997 | 0.9928 - 1.0000 | 0.946 | 0.971 | 0.946 | 0.971 | 0.946 | 0.946 | 0.946 | 0.5 |
|  | DECT_habitat_d-test | 0.304 | 0.353 | 0.1842 - 0.5221 | 0.5 | 0.2 | 0.25 | 0.429 | 0.25 | 0.5 | 0.333 | 0.2 |
|  | DECT_habitat_d-val | 0.514 | 0.501 | 0.3546 - 0.6483 | 0.381 | 0.571 | 0.276 | 0.683 | 0.276 | 0.381 | 0.32 | 0.4 |
|  | DECT_habitats-train | 0.981 | 1 | 1.0000 - 1.0000 | 0.946 | 1 | 1 | 0.971 | 1 | 0.946 | 0.972 | 5.00E-01 |
|  | DECT_habitats-test | 0.739 | 0.729 | 0.5464 - 0.9120 | 0.312 | 0.967 | 0.833 | 0.725 | 0.833 | 0.312 | 0.455 | 6.00E-01 |
|  | DECT_habitats-val | 0.729 | 0.628 | 0.4786 - 0.7779 | 0.143 | 0.98 | 0.75 | 0.727 | 0.75 | 0.143 | 0.24 | 7.00E-01 |
|  | DECT_radiomics-train | 0.981 | 0.999 | 0.9969 - 1.0000 | 0.946 | 1 | 1 | 0.971 | 1 | 0.946 | 0.972 | 0.5 |
|  | DECT_radiomics-test | 0.674 | 0.645 | 0.4622 - 0.8274 | 0.125 | 0.967 | 0.667 | 0.674 | 0.667 | 0.125 | 0.211 | 0.6 |
|  | DECT_radiomics-val | 0.757 | 0.719 | 0.5807 - 0.8575 | 0.238 | 0.98 | 0.833 | 0.75 | 0.833 | 0.238 | 3.70E-01 | 0.6 |
| **SVM** | CT_habitat_a-train | 0.945 | 0.949 | 0.8982 - 1.0000 | 0.878 | 0.987 | 0.977 | 0.929 | 0.977 | 0.878 | 0.925 | 0.606 |
|  | CT_habitat_a-test | 0.714 | 0.762 | 0.6320 - 0.8921 | 0.636 | 0.765 | 0.636 | 0.765 | 0.636 | 0.636 | 0.636 | 0.316 |
|  | CT_habitat_a-val | 0.732 | 0.715 | 0.5896 - 0.8397 | 0.633 | 0.805 | 0.704 | 0.75 | 0.704 | 0.633 | 0.667 | 0.383 |
|  | CT_habitat_b-train | 0.898 | 0.929 | 0.8749 - 0.9830 | 0.857 | 0.924 | 0.875 | 0.912 | 0.875 | 0.857 | 0.866 | 0.396 |
|  | CT_habitat_b-test | 0.661 | 0.687 | 0.5440 - 0.8303 | 0.636 | 0.676 | 0.56 | 0.742 | 0.56 | 0.636 | 0.596 | 0.425 |
|  | CT_habitat_b-val | 0.704 | 0.637 | 0.4947 - 0.7785 | 0.433 | 0.902 | 0.765 | 0.685 | 0.765 | 0.433 | 0.553 | 0.625 |
|  | CT_habitats-train | 0.945 | 0.981 | 0.9536 - 1.0000 | 0.939 | 0.949 | 0.92 | 0.962 | 0.92 | 0.939 | 0.929 | 0.26 |
|  | CT_habitats-test | 0.75 | 0.791 | 0.6651 - 0.9178 | 0.727 | 0.765 | 0.667 | 0.812 | 0.667 | 0.727 | 0.696 | 0.472 |
|  | CT_habitats-val | 0.718 | 0.728 | 0.6050 - 0.8502 | 0.5 | 0.878 | 0.75 | 0.706 | 0.75 | 0.5 | 0.6 | 0.612 |
|  | CT_radiomics-train | 0.914 | 0.973 | 0.9515 - 0.9952 | 0.878 | 0.937 | 0.896 | 0.925 | 0.896 | 0.878 | 0.887 | 0.315 |
|  | CT_radiomics-test | 0.732 | 0.789 | 0.6693 - 0.9083 | 0.773 | 0.706 | 0.63 | 0.828 | 0.63 | 0.773 | 0.694 | 0.387 |
|  | CT_radiomics-val | 0.704 | 0.735 | 0.6123 - 0.8576 | 0.7 | 0.707 | 0.636 | 0.763 | 0.636 | 0.7 | 0.667 | 0.37 |
|  | DECT_habitat_c-train | 0.905 | 0.971 | 0.9364 - 1.0000 | 0.919 | 0.897 | 0.829 | 0.953 | 0.829 | 0.919 | 0.872 | 0.209 |
|  | DECT_habitat_c-test | 0.739 | 0.773 | 0.6184 - 0.9275 | 0.687 | 0.767 | 0.611 | 0.821 | 0.611 | 0.687 | 0.647 | 0.348 |
|  | DECT_habitat_c-val | 0.643 | 0.776 | 0.6529 - 0.8981 | 0.905 | 0.531 | 0.452 | 0.929 | 0.452 | 0.905 | 0.603 | 0.256 |
|  | DECT_habitat_d-train | 0.819 | 0.743 | 0.6271 - 0.8594 | 0.541 | 0.971 | 0.909 | 0.795 | 0.909 | 0.541 | 0.678 | 0.424 |
|  | DECT_habitat_d-test | 0.674 | 0.45 | 0.2561 - 0.6439 | 0.125 | 0.967 | 0.667 | 0.674 | 0.667 | 0.125 | 0.211 | 0.541 |
|  | DECT_habitat_d-val | 0.586 | 0.67 | 0.5381 - 0.8011 | 0.714 | 0.531 | 0.395 | 0.812 | 0.395 | 0.714 | 0.508 | 0.293 |
|  | DECT_habitats-train | 0.933 | 0.965 | 0.9185 - 1.0000 | 0.892 | 0.956 | 0.917 | 0.942 | 0.917 | 0.892 | 0.904 | 2.90E-01 |
|  | DECT_habitats-test | 0.696 | 0.769 | 0.6303 - 0.9072 | 0.625 | 0.733 | 0.556 | 0.786 | 0.556 | 0.625 | 0.588 | 4.28E-01 |
|  | DECT_habitats-val | 0.871 | 0.88 | 0.7719 - 0.9890 | 0.714 | 0.939 | 0.833 | 0.885 | 0.833 | 0.714 | 0.769 | 5.90E-01 |
|  | DECT_radiomics-train | 0.914 | 0.947 | 0.8937 - 0.9998 | 0.838 | 0.956 | 0.912 | 0.915 | 0.912 | 0.838 | 0.873 | 0.425 |
|  | DECT_radiomics-test | 0.848 | 0.84 | 0.7065 - 0.9727 | 0.875 | 0.833 | 0.737 | 0.926 | 0.737 | 0.875 | 0.8 | 0.311 |
|  | DECT_radiomics-val | 0.614 | 0.747 | 0.6211 - 0.8735 | 0.905 | 0.49 | 0.432 | 0.923 | 0.432 | 0.905 | 5.85E-01 | 0.175 |
| **XGBoost** | CT_habitat_a-train | 0.992 | 1 | 1.0000 - 1.0000 | 0.98 | 1 | 1 | 0.987 | 1 | 0.98 | 0.99 | 0.565 |
|  | CT_habitat_a-test | 0.732 | 0.725 | 0.5813 - 0.8679 | 0.5 | 0.882 | 0.733 | 0.732 | 0.733 | 0.5 | 0.595 | 0.503 |
|  | CT_habitat_a-val | 0.662 | 0.663 | 0.5284 - 0.7968 | 0.567 | 0.732 | 0.607 | 0.698 | 0.607 | 0.567 | 0.586 | 0.362 |
|  | CT_habitat_b-train | 0.977 | 0.999 | 0.9976 - 1.0000 | 0.98 | 0.975 | 0.96 | 0.987 | 0.96 | 0.98 | 0.97 | 0.448 |
|  | CT_habitat_b-test | 0.661 | 0.666 | 0.5193 - 0.8136 | 0.5 | 0.765 | 0.579 | 0.703 | 0.579 | 0.5 | 0.537 | 0.383 |
|  | CT_habitat_b-val | 0.563 | 0.593 | 0.4597 - 0.7272 | 0.767 | 0.415 | 0.489 | 0.708 | 0.489 | 0.767 | 0.597 | 0.22 |
|  | CT_habitats-train | 0.992 | 1 | 1.0000 - 1.0000 | 0.98 | 1 | 1 | 0.987 | 1 | 0.98 | 0.99 | 0.642 |
|  | CT_habitats-test | 0.696 | 0.622 | 0.4622 - 0.7812 | 0.364 | 0.912 | 0.727 | 0.689 | 0.727 | 0.364 | 0.485 | 0.637 |
|  | CT_habitats-val | 0.662 | 0.657 | 0.5263 - 0.7867 | 0.633 | 0.683 | 0.594 | 0.718 | 0.594 | 0.633 | 0.613 | 0.444 |
|  | CT_radiomics-train | 0.992 | 1 | 1.0000 - 1.0000 | 0.98 | 1 | 1 | 0.987 | 1 | 0.98 | 0.99 | 0.582 |
|  | CT_radiomics-test | 0.768 | 0.758 | 0.6200 - 0.8961 | 0.636 | 0.853 | 0.737 | 0.784 | 0.737 | 0.636 | 0.683 | 0.517 |
|  | CT_radiomics-val | 0.746 | 0.727 | 0.6023 - 0.8522 | 0.667 | 0.805 | 0.714 | 0.767 | 0.714 | 0.667 | 0.69 | 0.31 |
|  | DECT_habitat_c-train | 0.99 | 1 | 1.0000 - 1.0000 | 0.973 | 1 | 1 | 0.986 | 1 | 0.973 | 0.986 | 0.608 |
|  | DECT_habitat_c-test | 0.652 | 0.752 | 0.6079 - 0.8963 | 0.875 | 0.533 | 0.5 | 0.889 | 0.5 | 0.875 | 0.636 | 0.168 |
|  | DECT_habitat_c-val | 0.557 | 0.675 | 0.5436 - 0.8072 | 0.81 | 0.449 | 0.386 | 0.846 | 0.386 | 0.81 | 0.523 | 0.223 |
|  | DECT_habitat_d-train | 0.981 | 0.998 | 0.9938 - 1.0000 | 0.946 | 1 | 1 | 0.971 | 1 | 0.946 | 0.972 | 0.416 |
|  | DECT_habitat_d-test | 0.457 | 0.481 | 0.3050 - 0.6575 | 0.812 | 0.267 | 0.371 | 0.727 | 0.371 | 0.812 | 0.51 | 0.151 |
|  | DECT_habitat_d-val | 0.7 | 0.476 | 0.3150 - 0.6374 | 0.143 | 0.939 | 0.5 | 0.719 | 0.5 | 0.143 | 0.222 | 0.844 |
|  | DECT_habitats-train | 0.99 | 1 | 1.0000 - 1.0000 | 0.973 | 1 | 1 | 0.986 | 1 | 0.973 | 0.986 | 5.41E-01 |
|  | DECT_habitats-test | 0.804 | 0.797 | 0.6526 - 0.9412 | 0.75 | 0.833 | 0.706 | 0.862 | 0.706 | 0.75 | 0.727 | 2.06E-01 |
|  | DECT_habitats-val | 0.743 | 0.779 | 0.6550 - 0.9028 | 0.667 | 0.776 | 0.56 | 0.844 | 0.56 | 0.667 | 0.609 | 4.27E-01 |
|  | DECT_radiomics-train | 0.99 | 1 | 1.0000 - 1.0000 | 0.973 | 1 | 1 | 0.986 | 1 | 0.973 | 0.986 | 0.679 |
|  | DECT_radiomics-test | 0.739 | 0.779 | 0.6410 - 0.9173 | 0.812 | 0.7 | 0.591 | 0.875 | 0.591 | 0.812 | 0.684 | 0.202 |
|  | DECT_radiomics-val | 0.629 | 0.689 | 0.5547 - 0.8233 | 0.762 | 0.571 | 0.432 | 0.848 | 0.432 | 0.762 | 5.52E-01 | 0.174 |

**Table S5:** Center name, CT equipment, and scanning parameters.

|  | **Name** | **CT equipment** | **NCCT and CTA** | **Contrast agent** |
| --- | --- | --- | --- | --- |
| **Center A** | Shanghai Sixth People's Hospital Affiliated to Shanghai Jiao Tong University School of Medicine | Philips Brilliance ICT 64 slices | Tube voltage: 120 kV; tube current: 333 mA; reconstruction thickness: 1.0 mm; 0.67 mm | Bayer Ultravist 370; flow rate: 4 mL/s |
|  |  | Toshiba Aquilion PRIME 80 slices | Tube voltage: 120 kV; tube current: 300 mA; reconstruction thickness: 1.0 mm; 0.63 mm |  |
| **Center B** | Affiliated Hospital of Nantong University | Philips Brilliance ICT 256 slices | Tube voltage: 120 kV; tube current: 360 mA; reconstruction thickness: 1.0 mm; 0.67 mm | Bayer Ultravist 370; flow rate: 3–4 mL/s |
|  |  | Philips Brilliance ICT 64 slices | Tube voltage: 120 kV; tube current: 330 mA; reconstruction thickness: 1.0 mm; 0.67 mm | Hengrui Loversol 320; flow rate: 3–5 mL/s |
| **Center C** | Wuxi Second People's Hospital | Toshiba Aquilion ONE 320 slices | Tube voltage: 120 kV; tube current: 300 mA; reconstruction thickness: 1.0 mm; 0.63 mm | Hengrui Loversol 320; flow rate: 3–5 mL/s. |

**Appendix E2.** DECT was acquired on a scanner (SOMATOMForce; Siemens Healthineers) using two X-ray tubes in all centers. The settings for the scanners were as follows: collimation, 128 × 0.6 mm; rotation time, 0.25 s; pitch, 0.7; reference tube current-time product, 90 mAs for the 90-kVp tube and 69 mAs for the Sn150-kVp tube; reformatted section thickness, 1.0 mm; and reformatted section increment, 0.7 mm. The enrolled patients were administered iodi-nated contrast media (Ultravist 370; Bayer Schering Pharma) through the ulnar vein (1.5 mL/kg and 5 mL/s), followed by a bolus injection of 30 mL of saline. The raw DECT data (including high- and low-keV data) were imported into the post-processing workstation (syngo. via VB20A, Dual Energy; Siemens Healthineers), recon-structed, and exported as mixed images and VM (40 keV and 190 keV), VNC, and iodine concentration (IC) images. CT perfusion scans covered the same anatomical range, with a tube voltage of 80 kV, tube current of 90 mAs, and gantry rotation time of 0.25 s/r, as well as 27 scans with an average scan time of 1.5 s per scan.

**Table S6:** List of the radiomic features (*n* = 107).

| **Firstorder**  **(n=18)** | 10Percentile;90Percentile;Energy;Entropy;InterquartileRange;Kurtosis;Maximum;Mean;MeanAbsoluteDeviation;Median;Minimum;Range;RobustMeanAbsoluteDeviation;RootMeanSquared;Skewness;TotalEnergy;Uniformity;Variance |
| --- | --- |
| **Shape**  **(n=14)** | Elongation;Flatness;LeastAxisLength;MajorAxisLength;Maximum2DdiameterColumn;Maximum2DdiameterRow;Maximum2DdiameterSlice;Maximum3Ddiameter;MeshVolume;MinorAxisLength;Sphericity;SurfaceArea;SurfaceVolumeRatio;VoxelVolume |
| **GlLDM**  **(n=14)** | DependenceEntropy;DependenceNonUniformity;DependenceNonUniformityNormalized;DependenceVariance;GrayLevelNonUniformity;GrayLevelVariance;HighGrayLevelEmphasis;LargeDependenceEmphasis;LargeDependenceHighGrayLevelEmphasis;LargeDependenceLowGrayLevelEmphasis;LowGrayLevelEmphasis;SmallDependenceEmphasis;SmallDependenceHighGrayLevelEmphasis;SmallDependenceLowGrayLevelEmphasis |
| **GLRLM**  **(n=16)** | GrayLevelNonUniformity;GrayLevelNonUniformityNormalized;GrayLevelVariance;HighGrayLevelRunEmphasis;LongRunHighGrayLevelEmphasis;LongRunLowGrayLevelEmphasis;LowGrayLevelRunEmphasis;RunEntropy;RunLengthNonUniformity;RunLengthNonUniformityNormalized;RunPercentage;RunVariance;ShortRunEmphasis;ShortRunHighGrayLevelEmphasis;ShortRunLowGrayLevelEmphasis |
| **GLSZM**  **(n=16)** | GrayLevelNonUniformity;GrayLevelNonUniformityNormalized;GrayLevelVariance;HighGrayLevelZoneEmphasisLargeAreaEmphasis;LargeAreaHighGrayLevelEmphasis;LargeAreaLowGrayLevelEmphasis;LowGrayLevelZoneEmphasis;SizeZoneNonUniformity;SizeZoneNonUniformityNormalized;SmallAreaEmphasis;SmallAreaHighGrayLevelEmphasis;SmallAreaLowGrayLevelEmphasis;ZoneEntropy;ZonePercentage;ZoneVariance |
| **NGTDM**  **(n=5)** | Busyness;Coarseness;Complexity;Contrast;Strength |
